# Biomimetic Scaffolds Modulate the Posttraumatic Inflammatory Response in Articular Cartilage Contributing to Enhanced Neoformation of Cartilaginous Tissue In Vivo

**DOI:** 10.1002/adhm.202101127

**Published:** 2021-10-28

**Authors:** Guillermo Bauza‐Mayol, Marcos Quintela, Ava Brozovich, Michael Hopson, Shazad Shaikh, Fernando Cabrera, Aaron Shi, Federica Banche Niclot, Francesca Paradiso, Emman Combellack, Tom Jovic, Paul Rees, Ennio Tasciotti, Lewis W. Francis, Patrick Mcculloch, Francesca Taraballi

**Affiliations:** ^1^ Center for Musculoskeletal Regeneration Houston Methodist Research Institute 6670 Bertner Ave. Houston TX 77030 USA; ^2^ Orthopedics & Sports Medicine Houston Methodist Hospital 6550 Fannin St. Houston TX 77030 USA; ^3^ Reproductive Biology and Gynaecological Oncology Group Swansea University Medical School Singleton Park Swansea SA2 8PP UK; ^4^ Texas A&M College of Medicine Bryan TX 77807 USA; ^5^ Reconstructive Surgery and Regenerative Medicine Research Group Swansea University Medical School Singleton Park Swansea SA2 8PP UK; ^6^ Polytechnic of Turin Department of Applied Science and Technology Corso Duca degli Abruzzi 24 Torino 10129 Italy; ^7^ IRCCS San Raffaele Pisana Via della Pisana 235 Rome 00163 Italy

**Keywords:** cartilage repair, osteoarthritis, scaffolds, tissue engineering

## Abstract

Focal chondral lesions of the knee are the most frequent type of trauma in younger patients and are associated with a high risk of developing early posttraumatic osteoarthritis. The only current clinical solutions include microfracture, osteochondral grafting, and autologous chondrocyte implantation. Cartilage tissue engineering based on biomimetic scaffolds has become an appealing strategy to repair cartilage defects. Here, a chondrogenic collagen‐chondroitin sulfate scaffold is tested in an orthotopic Lapine in vivo model to understand the beneficial effects of the immunomodulatory biomaterial on the full chondral defect. Using a combination of noninvasive imaging techniques, histological and whole transcriptome analysis, the scaffolds are shown to enhance the formation of cartilaginous tissue and suppression of host cartilage degeneration, while also supporting tissue integration and increased tissue regeneration over a 12 weeks recovery period. The results presented suggest that biomimetic materials could be a clinical solution for cartilage tissue repair, due to their ability to modulate the immune environment in favor of regenerative processes and suppression of cartilage degeneration.

## Graphical abstract

A biomimetic immune tuning scaffold is implanted in a full‐thickness chondral defect in rabbits. The early inflammatory infiltrates changed in the treated animals resulting in a better cartilage homeostasis at longer timepoints. This work represents the first approach of tuning the inflammatory reaction with a bulk material to achieve a better regeneration in cartilage.

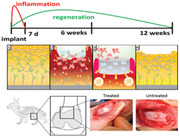

## 1 Introduction

Focal chondral lesions of the knee are the most frequent trauma in younger patients^[^ ^1^ ^]^ and are associated with a high risk of developing early posttraumatic osteoarthritis (OA).^[^ ^2^, ^3^ ^]^ However, there are limitations on articular cartilage regeneration due to a lack of vasculature, lymphatic, and nerve supply.^[^ ^4^ ^]^ As a result, stabilization surgery is required in most clinical cases in order to relieve the pain associated with the chondral lesion/trauma, as well as to enhance functional recovery.^[^ ^5^ ^]^

Current treatments for articular cartilage lesions include microfracture, osteochondral grafting, and autologous chondrocyte implantation (ACI). These techniques however have several drawbacks. While ACI has the limitation of site morbidity and cartilage tissue availability,^[^ ^6^ ^]^ allografts may potentially elicit immune responses.^[^ ^7^ ^]^ In addition, chondroplasty involves exposing subchondral bone or layers of cartilage, and the natural history of progression after treatment is unknown. Moreover, ACI requires full‐thickness cartilage at the margins around the defect and prolonged protection postoperatively is needed to allow for chondrocyte maturation.^[^ ^8^ ^]^ Although these techniques assure joint stabilization, more recently it is thought that OA may also develop in joints that have been successfully stabilized by surgery, that have normal biomechanics.^[^ ^8^ ^]^ Since traditional therapies assure pain reduction but do not truly restore the original cartilage homeostasis, tissue engineering represents the most promising tool to greatly improve outcomes following joint lesion development.^[^ ^9^ ^]^

There have been a few groups that have explored the use of immune modulation through tissue engineering.^[^ ^9^, ^10^ ^]^ However, it appears that the tissue engineering field has yet to apply these principles to joint trauma and concurrent OA development. For instance, OA has traditionally been classified as a noninflammatory disease. However, this view has been recently challenged as new research suggests the presence of ongoing immune processes’ within the OA joint and the synovium.^[^ ^5^, ^10^ ^]^ Moreover, OA disease management using anti‐inflammatory drugs and corticosteroids provides further evidence that there is an inflammatory component to the disease.^[^ ^11^ ^]^

Although the goal of tissue engineering has always been to boost the regeneration process at the site of the cartilage defect,^[^ ^12^ ^]^ recent considerations and knowledge related to the inflammatory nature of OA, could point towards the development of new solutions that not only improve the regeneration of lost tissue but also reduce the likelihood of developing late‐onset OA. In this regard, the pioneering work of the Elisseeff group^[^ ^13^ ^]^ has shown the possibility to conjugate a scaffold with an anti‐inflammatory drug in order to reduce the activation of NF‐kB and the subsequent inflammatory cascade. Our group has recently developed a biomimetic scaffold that we have shown to have not only anti‐inflammatory effects,^[^ ^14^ ^]^ but also chondrogenic potential in vitro.^[^ ^15^ ^]^ These properties are both linked to the specific composition, architecture, and mechanical features of the scaffold.

Following injury, inflammation occurs at the site of the joint, and we believe that by tuning the inflammatory reaction, an immune‐modulatory implant can favor cartilage homeostasis. Most recently we have shown that the chondroitin sulfate (CS) functionalized collagen scaffolds (CLCS) used in a 3D in vitro culture model demonstrate the ability to modulate the inflammatory cascade while housing both chondrocyte progenitor and bone marrow‐derived mesenchymal stem cells (MSCs).^[^ ^16^ ^]^ Following these promising results, in this study, we aimed to understand how an immune‐modulatory scaffold helps to restore cartilage homeostasis after trauma, indicated by articular cartilage regeneration.

This study aimed to assess the ability of the CLCS to treat full‐thickness chondral defects in vivo. Using a combination of delayed gadolinium‐enhanced MRI of cartilage (dGEMRIC), CyTOF, total RNA sequencing, and standard histological analysis, we monitored the effect of our CLCS immuno‐tuning scaffold material for cartilage regeneration following defect introduction. Activated gene expression cascades indicate the potential regulation of transcriptomic networks consistent with modified inflammation, extracellular matrix (ECM)–cell communication, and renewed cartilage homeostasis at 12 weeks postimplantation. Our tissue engineering design displays a new proof of concept to manage OA using a scaffold material that is safe for use in humans. If used clinically, it could provide a regenerative treatment approach to a disease with limited treatment options.

## 2 Results

The porous structure of CSCL scaffolds after freeze‐drying has been determined by SEM imaging (Figure S1A, Supporting Information). At lower magnification, the sample structure is composed by interconnected pores with boundaries defined by sheet‐like structure of fibrillar collagen. At higher magnification, it can be appreciated how the presence of chondroitin sulfate resulted in a fibrous substructure (Figure S1B,C, Supporting Information). FTIR spectra reported in Figure S1D (Supporting Information) showed the characteristic collagen vibration peaks like Amide I (1700–1600 cm^−1^) and amide II (1600–1500 cm^−1^), related to the stretching vibration of C═O bonds and to C—N stretching and N—H bending vibration, respectively. The sample contained C ═ O, C—N, and N—H bonds. Amide III region (approximately 1200–1300 cm^−1^) is related to the C—N and C—C stretching, N—H bonds, and CH2 wagging from the glycine backbone and proline side chain.

**Figure 1:**
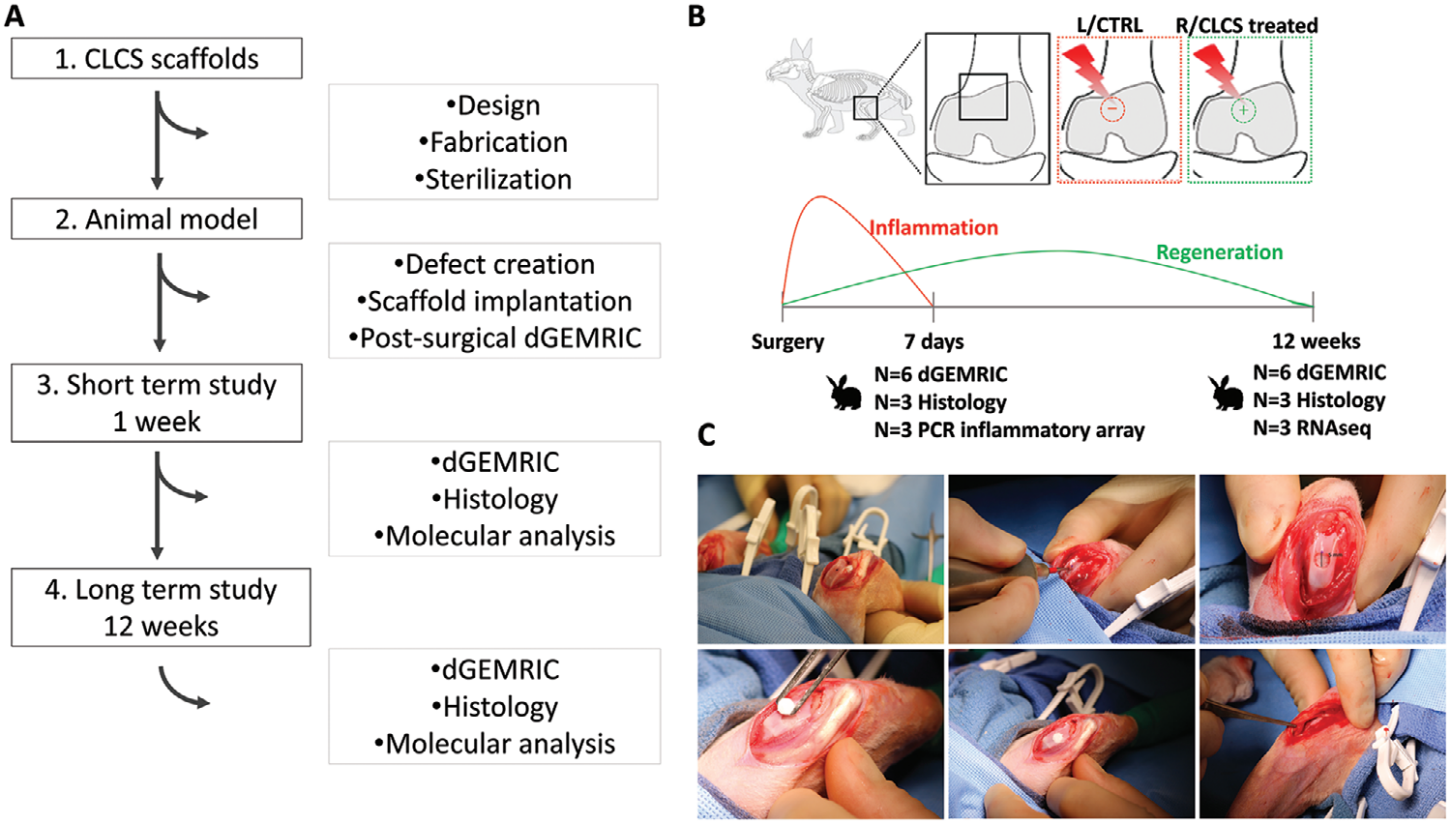
In vivo *s*tudy design. A) Pipeline of articular cartilage regeneration in vivo. B) Defects were created in the trochlear groove of NZW rabbits and treated with biomimetic CLCS scaffolds or left untreated. The molecular composition and pathway analysis were evaluated at 7 days, 6 and 12 weeks respectively to assess inflammation resolution and regeneration. C) Cartilage defect model surgical procedure from left to right top to bottom. Right and left knee joints opened with lateral incision and manual lateral patellar dislocation followed by knee flexion to expose the femoral condyles. Creation of the surgical defect 5 mm diameter by 1 mm depth using a surgical drill. Cartilage full‐thickness defect. Positioning of the CLCS scaffold in the created defect without external attachments. Initiation of the manual patella repositioning with the leg in extension, visual inspection of the surgical procedure after patellar reposition and successive joint flexion and extensions to regain correct mechanics of the join and maintain the CLCS scaffold under the patella.

Compressive tests were carried out to evaluate the compressive strength and stiffness of the scaffold (Figure S1E, Supporting Information). The machine was set up with an appropriate loading cells of 10 N, the scaffold placed between two steel plates, and the force record for 60 s to a strain level of approximately 35%. The results summarized in Figure 1E (Supporting Information) show very weak resistance to compression as expected for spongy scaffolds^[^ ^17^, ^18^ ^]^ Shear rheometry is currently gaining interests as a diagnostic tool to quantify the mechanical properties of soft tissues, since it allows evaluation of entire tissue volume mechanic instead of just a thin surface layer of cells performed by other techniques like atomic force microscopy.^[^ ^15^ ^]^ In this work, we use rheometric analysis to evaluate the mechanical parameters of the whole CSCL scaffold (Figure 1F, Supporting Information). The rheometer system was optimized to characterize the elastic, elastoplastic and viscous flow behavior of the scaffold. Scaffold storage modulus and loss modulus as a function of frequency at 1 Hz was recorded. CSCL storage modulus (*G*′) was 35244 ± 9439.18 kPa and loss modulus (*G*″) 10728.5 ± 3462.967 kPa, confirming a high elastic behavior of the scaffold used for this work (*G*′ ≥ *G*).

An experimental procedure was performed with the utilization of the CLCS scaffold implanted into a critical size defect in vivo model at an orthotopic site in a lapine joint.^[^ ^19^ ^]^ A pipeline of the regeneration study is shown in **Figure** **1**A,B. New Zealand rabbits underwent a surgical procedure to generate a critical size defect at the trochlear groove of the knee joint. A 5 mm diameter by 1 mm height CLCS scaffold was implanted at the right leg defect, while the left knee was left untreated and used as a control defect. The patella was then reduced and aligned by checking knee joint flexion mechanics, followed by fascial and skin closures performed using absorbable sutures. Images of the critical defects are shown in Figure 1C.

**Figure 2:**
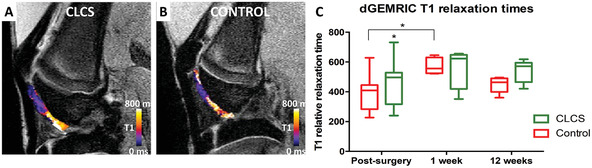
dGEMRIC noninvasive analysis. A,B) Empty defect (control) and CLCS scaffold treated representative dGEMRIC MRI images showing gadolinium contrast T1 relaxation times color coded post‐surgery and 12 weeks after treatment. C) CLCS treated cartilage T1 relaxation times compared to empty defect controls at different time points. Results were tested for significance using two‐way ANOVA followed by Sidak's multiple comparisons test between groups from a minimum of six biological replicates. * was used for *p* values lower than 0.05.

All rabbits were processed for post‐surgery dGEMRIC MRI in order to monitor the success of the surgery and create a baseline for the potential repair process. dGEMRIC is a non‐invasive technique that generates cartilage images that can be used as an indirect measurement of glycosaminoglycan (GAG) concentration in the extracellular cartilage matrix.^[^ ^20^ ^]^ Representative dGEMRIC MRI images and post‐surgical T1 quantification results are shown in **Figure** **2**.

The area where the defect was created can be clearly differentiated from the untouched cartilage zone displaying a brighter color coding (Figure 2A). Right and left knee T1 relaxation time images were then quantified resulting in 391.45 ± 113.34 s and 458.5 ± 136.37 s, respectively (Figure 2B). Interestingly, CLCS implanted right knees exhibited a significant increase (1.17 ± 0.35‐fold, *p* < 0.05) in T1 relaxation time in comparison to the untreated control.

Images and T1 relaxation times were quantified at 1 and 12 weeks postsurgery. CLCS T1 relaxation mapping displayed a greater area of colored coding and very few purple GAG depleted areas. In comparison, untreated controls exhibited less stained areas, showing higher heterogenicity and purple areas related to poor GAG content (Figure 2A). CLCS exhibited non‐significant differences in T1 relaxation time average at 1 and 12 week postsurgery respectively (563.58 ± 123.01 s and 538.43 ± 68.24 s) in comparison to untreated controls. Interestingly, untreated cartilage displayed a significant increase (1.46 ± 0.13‐fold, *p* < 0.05) in T1 relaxation time 1 week postsurgery in comparison to untreated postsurgery controls.

In order to corroborate the above data, cells were extracted from the excised CLCS scaffolds 1 week following implantation. Total RNA was extracted, and a gene expression profile was performed. The inflammatory pathways induced by CLCS implanted at damaged cartilage tissue in vivo were compared to untreated damaged tissue and healthy controls through an inflammatory cytokines and receptors PCR array^[^ ^21^ ^]^ (**Table** **1**).

**Table 1:** List of genes found over‐expressed and underexpressed among the 84 tested through the RT^2^ Profiler PCR array rabbit inflammatory cytokines & receptors in CSCL scaffolds in vivo compared to untreated defect and to healthy articular cartilage 1 week postimplantation

Symbol	Refseq	Description	Fold Change	95% CI
Differentially expressed genes in CSCL compared to empty defect				
*CXCR4*	XM_002712124	Chemokine (C‐X‐C motif) receptor 4	2.05	0.02
*IL7R*	XM_002714135	Interleukin 7 receptor	1.91	0.03
*ADIPOQ*	NM_001082222	Adiponectin, C1Q and collagen domain containing	5.04	0.002
*BMP2*	NM_001082650	Bone morphogenetic protein 2	‐8.59	0.01
*OSM*	XM_008274704	Oncostatin M‐like	‐42.79	0.008
*CX3CL1*	XM_002711541	Chemokine (C‐X3‐C motif) ligand 1	‐3.75	0.0001
*IL23A*	XM_002711079	Interleukin 23, alpha subunit	‐1.99	0.03
Differentially expressed genes in CLCS compared to healthy control				
*IL1A*	NM_001101684	Interleukin 1, alpha	2.89	0.03
*IL1RN*	NM_001082770	interleukin 1 receptor antagonist	3.53	0.002
*MIF*	XM_002722561	Macrophage migration inhibitory factor	2.02	0.01
*IL10RA*	XM_008249453	Interleukin 10 receptor, alpha‐like	2.1	0.001
*BMP4*	NM_001195723	Bone morphogenetic protein 4	‐2.89	0.008
*CXCL13*	XM_008267697	Chemokine (C‐X‐C motif) ligand 13	‐9.55	0.01
*NAMPT*	XM_002712033	Nicotinamide phosphoribosyltransferase‐like	‐1.73	0.007
*IL21*	XM_008267970	Interleukin 21‐like	‐21.02	0.04
*IL7*	XM_008255687	Interleukin 7‐like	‐2.82	0.03
*IL22*	XM_002711248	Interleukin 22	‐9.06	0.03
*LEPR*	XM_008265107	Leptin receptor‐like	‐10.78	0.03
*TNFSF10*	XM_002716426	Tumor necrosis factor (ligand) superfamily, member 10‐like	‐2.19	0.01
*SPP1*	NM_001082194	Secreted phosphoprotein 1	‐5.05	0.02

Seven genes were found to be differentially expressed in CLCS treated explants in comparison to damaged untreated cartilage. The anti‐inflammatory adipokine ADIPOQ and chemokine receptors CXCR4 and IL7R, as well as the anti‐inflammatory proteins IL1RA and IL10, were significantly up regulated (*p* = 0.002 and *p* = 0.001 respectively). Pro‐inflammatory cytokines IL23A, CX3CL1, BMP2 and OSM and signaling molecules CXCL13, IL7, IL21, IL22, TNFSF10, SPP1 and LEPR were found downregulated compared to the healthy control.

**Figure 3:**
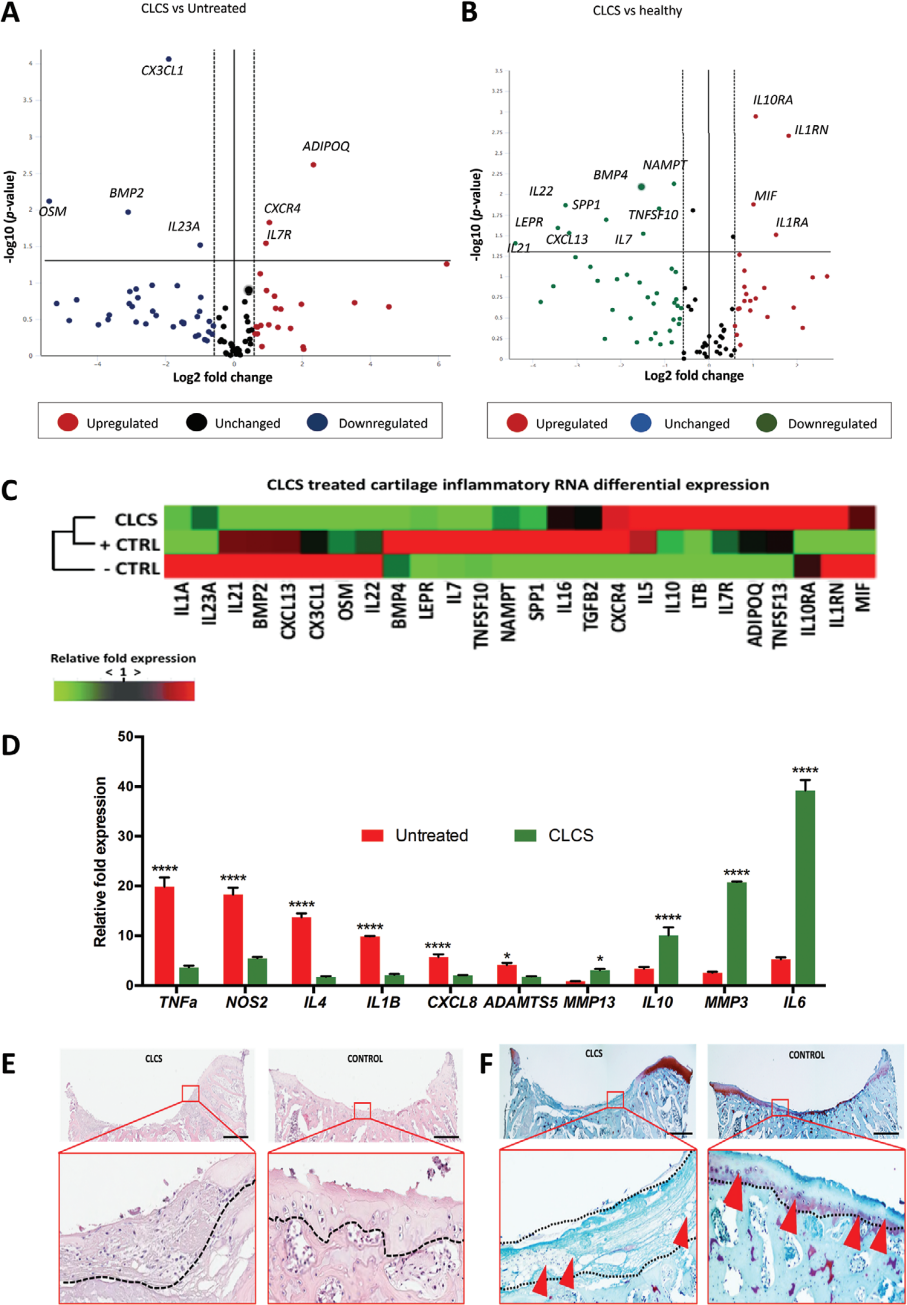
CLCS implanted scaffolds induction 1 week after surgery.A,B) Scatter volcano plots to visualize differential expression patterns tested through the RT^2^ Profiler PCR array rabbit inflammatory cytokines & receptors between CLCS treated and untreated control A) and between CLCS treated and B) healthy control cartilage. Vertical dotted lines correspond to 1.5‐fold up and down respectively, and the horizontal line represents a *p* value of 0.05. C) Heatmap of differentially expressed genes between hierarchically clustered CSCL treated, untreated (‐CTRL) and healthy cartilage (+CTRL) in in vivo explants. Differential expression levels of genes are displayed as color‐coded: red represents over‐expression while green under‐expression. D) Inflammatory pathway‐specific gene markers relative expression of CLCS treated tissue explants compared to untreated control explants 1‐week postimplantation (healthy control = 1 not shown). Results were tested for significance using two‐way ANOVA followed by Sidak's multiple comparisons test between groups from a minimum of three biological replicates. * and **** were used for *p* values lower than 0.05 and 0.0001. E,F) Representative images of E) hematoxylin and eosin staining of the cellular composition and structure and safranin‐O tissue staining F) of ECM composition. All images refer to untreated empty control (CONTROL) and CLCS scaffold (CLCS) treated joint defects (and insets in red boxes) at 7 d postsurgery. Scale bar represents 1 mm.

Volcano scatter and heatmap plots of differentially expressed genes between in vivo explants are shown in **Figure** **3**. Volcano plots display the *p* values in the vertical axis over the fold change expression in the horizontal axis. ADIPOQ was the most significantly overexpressed gene in CLCS treated cartilage compared to untreated, damaged control (Figure 3A). IL1RN and IL10RA displayed the highest level of significantly increased expression when compared to healthy cartilage control (Figure 3B). On the opposite site, IL21 expression displayed the most significant decrease in expression compared to the healthy controls. In addition, differentially expressed genes between the CL and CLCS in vivo explants were clustered in a heatmap of expression (Figure 3C). Interestingly, RNA expression profiles from CLCS treated explants clustered together with the healthy control explants and were opposing to the untreated, damaged cartilage explants.

To further evaluate the tissue molecular inflammatory response induced by CLCS scaffolds implanted at damaged articular cartilage, the RNA expression was evaluated using specific gene markers involved in inflammation within the cartilage.^[^ ^22^ ^]^ Cytokine expression including TNFa, NOS2, IL1B, IL4, IL6, CXCL8, and IL10 and cartilage proteases MMP3, MMP13, and ADAMTS5 specific primers were used to detect the level of expression in real‐time polymerase chain reaction (RT‐PCR) experiments (Figure 3D). CLCS treated explants exhibited a significant decrease (0.18 ± 0.01‐fold, *p* < 0.0001) in TNF*α* expression compared to untreated damaged control explants. Similarly, CLCS treated explants displayed a significant decrease (0.30 ± 0.01‐fold, *p* < 0.0001) in NOS2 expression. In addition, CLCS treated explants inflammatory cytokines IL1B, IL4, and CXCL8 RNA expression was found downregulated (*p* < 0.0001) in comparison to untreated damaged control. However, CLCS treated explants exhibited a significant increase (7.45 ± 0.34‐fold, *p* < 0.0001) in IL6 expression compared to untreated damaged control explants (Figure 3D).

Matrix proteases expression was also compared between groups. CLCS treated explants displayed a significant decrease (0.43 ± 0.01‐fold, *p* < 0.05) in ADAMTS5 expression compared to untreated damaged control. Differently, CLCS treated cartilage exhibited a significant increase (3.64 ± 0.26‐fold and 8.22 ± 0.05‐fold, *p* < 0.05 and *p* < 0.0001) in MMP13 and MMP3 expression respectively in comparison to untreated controls 1 d postsurgery. Interestingly, CLCS treated cartilage explants showed a significant increase (2.99 ± 0.40‐fold, *p* < 0.0001) in anti‐inflammatory IL10 expression compared to controls.

In order to determine the tissue level effect of these gene expression networks, CLCS treated and untreated explants were examined using H&E and Safranin O staining. H&E stained slides from 1 week postsurgical CLCS treated samples displayed nuclear and ECM staining, indicating the presence of cellular infiltrates at the damaged area (Figure 3E). The pink‐stained porous collagen‐based scaffolds could be delimited from the surrounding cartilage tissue. Interestingly, the CLCS scaffolds exhibited nuclei eosin staining indicating cellular infiltration from the surrounding tissues. In comparison, untreated defects displayed poor cellular presence (Figure 5E). Safranin O stained slides (Figure 3F) displayed dark nuclear staining confirming the cellular infiltration into the porous scaffolds. Moreover, CLCS treated samples lacked the presence of Safranin O stained areas, showing ECM depletion at the trauma site. The CLCS scaffold may be expected to stain red in the presence of Safranin O, however in some instances, monoclonal antibodies are required to stain proteoglycans following significant depletion.^[^ ^23^ ^]^ On the contrary, untreated defects displayed a continuous fade layer of reddish Safranin O stained area (probably the mineralized cartilage part remained after the defect) including the presence of round big cells neighbored close together (Figure 3F).

**Figure 4:**
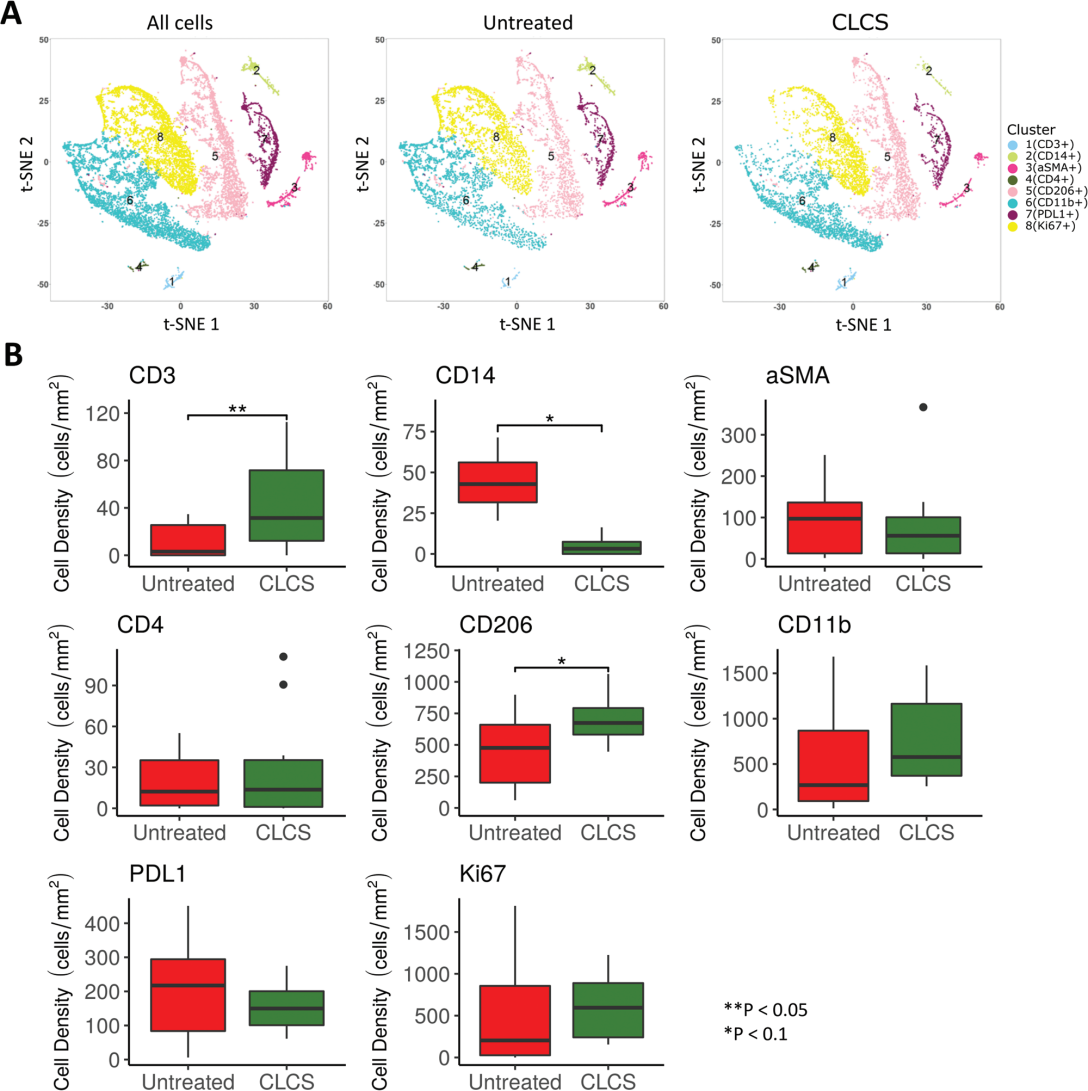
Immune cells’ infiltration after 1 week. A) Total data and empty defect (control) and scaffold treated tSNE plots constructed from Cytof imaging of inflammatory marker stain panels showing color‐coded CD marker‐based immune cell clusters, 1 week after treatment. B) Cell density values for each cluster, separated by ilastik and CellProfiler and attributed to the predominant maker. Results were tested for significance using a nonpaired t‐Test and significant differences in cell clusters denoted with an *. In order to determine the immunomodulary effects of CLCS scaffolds following injury, CyTOF was used to evaluate the infiltration of immune cells 1 week postimplant. Following implant, the CLCS scaffold has immediate effects on the type and number of immune cells that migrate and infiltrate to the site. Following implantation, sections of the knee joint were obtained and stained with different antibodies to determine relative prevalence of different cells. Figure 4B demonstrates that among untreated and CLCS samples, the relative prevalence of immune cells prevalent within the knee joint are different. CLCS samples had a higher proportion of CD^11b+^ and CD^206^ cells, markers that demonstrate immune cells that are more tissue regeneration rather than inflammation. When examining the cell density of different clusters, there is a statistically significant difference in a variety of immune cells; CLCS samples had a statistically higher number of CD^3+^ (*p* = 0.03) and CD^206+^ (*p* = 0.07) cells, but had a statistically lower number of CD^14+^ (*p* = 0.09), compared to untreated samples (Figure 4B). Moreover, when all CLCS samples are pooled, the clustering of immune cells is different compared to untreated samples (Figure 4A). Figure 4A demonstrates visually that CLCS samples have a smaller proportion in cluster 2 (CD^14+^) and cluster 8 (Ki^67+^).

In order to confirm the data of the RNA array, CyTOFwas used to evaluate the infiltration of immune cells 1 week postimplantation. Following implant, the CLCS scaffold has immediate effects on the type of immune cells that migrate and infiltrate to the site. **Figure** **4** demonstrates that among untreated and CLCS samples, the relative prevalence of immune cells within the knee joint are significantly different. CLCS samples had a higher proportion of CD^11b+^ and CD206 cells, markers that demonstrate immune cells that are more involved in inflammation resolution. When examining the cell density of different clusters, there is a statistically significant difference in a variety of immune cells; CLCS samples had a statistically higher number of CD^3+^ (*p* = 0.03) and CD^206+^ (*p* = 0.07) cells, but had a statistically lower number of CD^14+^ (*p* = 0.09), compared to untreated samples (Figure 4B). Moreover, when all CLCS samples are pooled, the clustering of immune cells is different compared to untreated samples (Figure 4A). Figure 4A demonstrates visually that CLCS samples have a smaller proportion in cluster 2 (CD^14+^) and cluster 8 (Ki^67+^).

**Figure 5:**
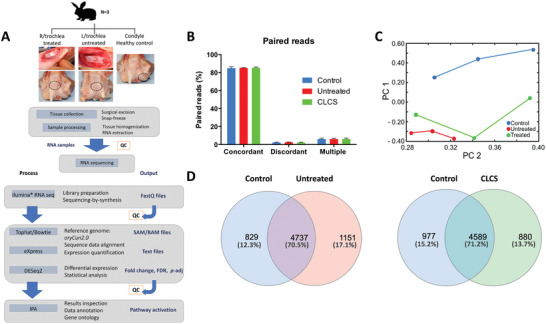
RNA sequencing 12 weeks postsurgery. A) Study design and pipeline analysis. CLCS scaffolds treated trochlear defects were excised surgically 12 weeks after surgery and compared to untreated defect and healthy cartilage controls. RNA was extracted from tissue samples and sequenced using Ilumina RNA sequencing technology. Data were aligned to “oryCun2.0” using Bowtie to produce SAM files. Unique alignments were then counted using eXpress to produce Text files. Using DESeq2, data were normalized prior to calculation of differential gene expression. IPA software was used to annotate the results obtained. L (left), R (right), QC (quality control). B) Quality control of the alignment to “oryCun2.0” displaying concordant discordant and multiple alignment percentages. C) PCA plot displaying the similarities correlated to the distances between experimental groups normalized according to gene expression. D) Venn diagrams showing the overlap between the lists of expressed transcripts in control, untreated, and CLCS cells.

Finally, in order to monitor the cartilage repair process at a molecular level, animals were sacrificed, and tissue was harvested at 12 weeks post surgery; CLCS treated, untreated and healthy cartilage tissues were then processed for RNA‐sequencing analysis (**Figure** **5**A). Prior to alignment, FastQC was used to monitor the quality of raw Illumina reads, all of which displayed acceptable QC metrics. High‐quality reads were unequivocally aligned to the reference genome, and multiple alignments were disregarded following recommended ENCODE Guidelines (**Table** **2** and Figure 5B).^[^ ^24^ ^]^

**Table 2:** RNA sequencing quality control parameters prior to data analysis. The table reports the sequencing data of the experimental samples number of reads in the right direction, left direction, and paired reads, the % alignment to the reference genome oryCun2a.fa, multiple and discordant alignments

Sample	Reads	Right reads		Left reads		Aligned pairs			
		Mapped	Multiple alignments	Mapped	Multiple alignments	Paired reads	Concordant	Discordant	Multiple
Control 1	59616857	90.40%	4.90%	89.80%	4.90%	51987157	85.50%	1.90%	4.90%
Control 2	61583331	88.50%	6.50%	87.90%	6.50%	52360874	83.10%	2.30%	6.50%
Control 3	60644061	91.50%	6.90%	90.80%	6.90%	53498659	86.00%	2.50%	6.90%
Untreated 1	70829783	90.90%	5.60%	90.10%	5.60%	62037139	85.60%	2.20%	5.60%
Untreated 2	62623989	91.20%	6.90%	90.20%	6.90%	54847093	85.20%	2.80%	7.00%
Untreated 3	68335605	90.80%	5.50%	89.70%	5.60%	59555514	85.00%	2.40%	5.60%
CLCS 1	59156456	90.20%	5.90%	88.90%	5.90%	51135627	84.50%	2.20%	6.00%
CLCS 2	67797552	90.90%	7.20%	89.70%	7.30%	59146477	85.20%	2.40%	7.30%
CLCS 3	67854294	91.10%	5.10%	90.10%	5.10%	59582743	86.40%	1.60%	5.20%

**Figure 6:**
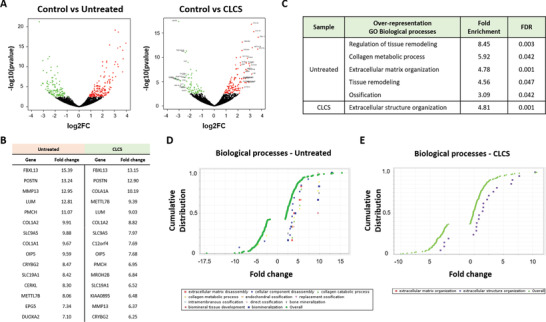
Cartilage transcriptome pathway analysis. A) Volcano plots comparing statistical significance against log2 fold change between control and untreated/CLCS samples. Green color dots represent significantly down‐regulated genes (log_2_FC < = ‐0.3, FDR < 0.05). Red color dots represent significantly upregulated genes (logFC > = 0.39, FDR <0.05). B) Top 15 genes altered (up) in untreated/CLCS cells compared with the control. C) Table containing the list of PANTHER pathways identified as over‐represented when comparing the lists of genes expressed in untreated/CLCS samples as opposed to control (PANTHER Annotation “GO Biological process”). D,E) Cumulative distribution analyses of the differentially expressed genes in untreated/CLCS cells, the graphs display fold‐change distributions of significantly enriched processes.

Sample replicates displayed significant reproducibility in terms of gene expression, as inferred from the principal component analyses (PCA) (Figure 5C). Interestingly, CLCS treated cartilage repaired for 12 weeks positioned somewhere in between both controls, suggesting a more efficient transition towards a healthy cartilage state (Figure 5C). The sequencing analysis of the healthy control sample identified 5566 different transcripts, while 5888 and 5469 transcripts were identified in the untreated and CLCS samples respectively (Figure 5D). Overall, transcript expression levels seemed consistent among specimens, however the untreated sample showed a modest increase in the percentage of unique transcripts as opposed to control in comparison with the CLCS sample (17.1% > 13.7%, **Figure** **6**D). These differences may account for increased transcriptional activity related to continuous wound healing.

In order to investigate the level of differential expression between samples (false discovery rate (FDR)<0.05), both untreated and CLCS expression profiles were compared to healthy cartilage using the DESeq2 software package. Results of differential gene expression analysis between healthy control and untreated samples pinpointed 293 genes with significantly different levels of expression (Figure 6A); 169 upregulated and 124 downregulated (Table S3, Supporting Information). Similarly, 198 genes significantly changed their expression levels when evaluating the differential expression levels of healthy control and CLCS samples (Figure 6A); 125 and 73 genes with up‐regulated and down‐regulated expression respectively (Table S3, Supporting Information). The list of top differentially expressed genes is depicted in Figure 6B and includes well‐known markers of tissue remodeling and extracellular matrix organization such as MMP13 and COL1A2 (Figure 6B).

To gain insight into which processes or pathways were significantly enriched within the list of differentially expressed transcripts, we performed overrepresentation and enrichment tests using the evolutionary relationship platform PANTHER. Interestingly, overrepresentation analyses on the genes differentially expressed in the untreated sample compared to control identified a series of enriched pathways that include processes such as “Regulation of tissue remodeling” or “Ossification” (Figure 6C). On the other hand, the only pathway that was significantly enriched upon cartilage growth on CLCS was “Extracellular structure organization” (Figure 6C). These results suggest the prevalence of remodeling features on the untreated wound at twelve weeks post‐injury, while the addition of CLCS seems to significantly abbreviate the duration of recovery. To corroborate the results observed with over‐representation analyses, we performed gene‐set enrichment tests using PANTHER software. These analyses confirmed that processes related with collagen metabolism and ossification, amongst others, were strongly enriched in the untreated sample (Figure 6D) but not in the CLCS sample (Figure 6E).

**Figure 7:**
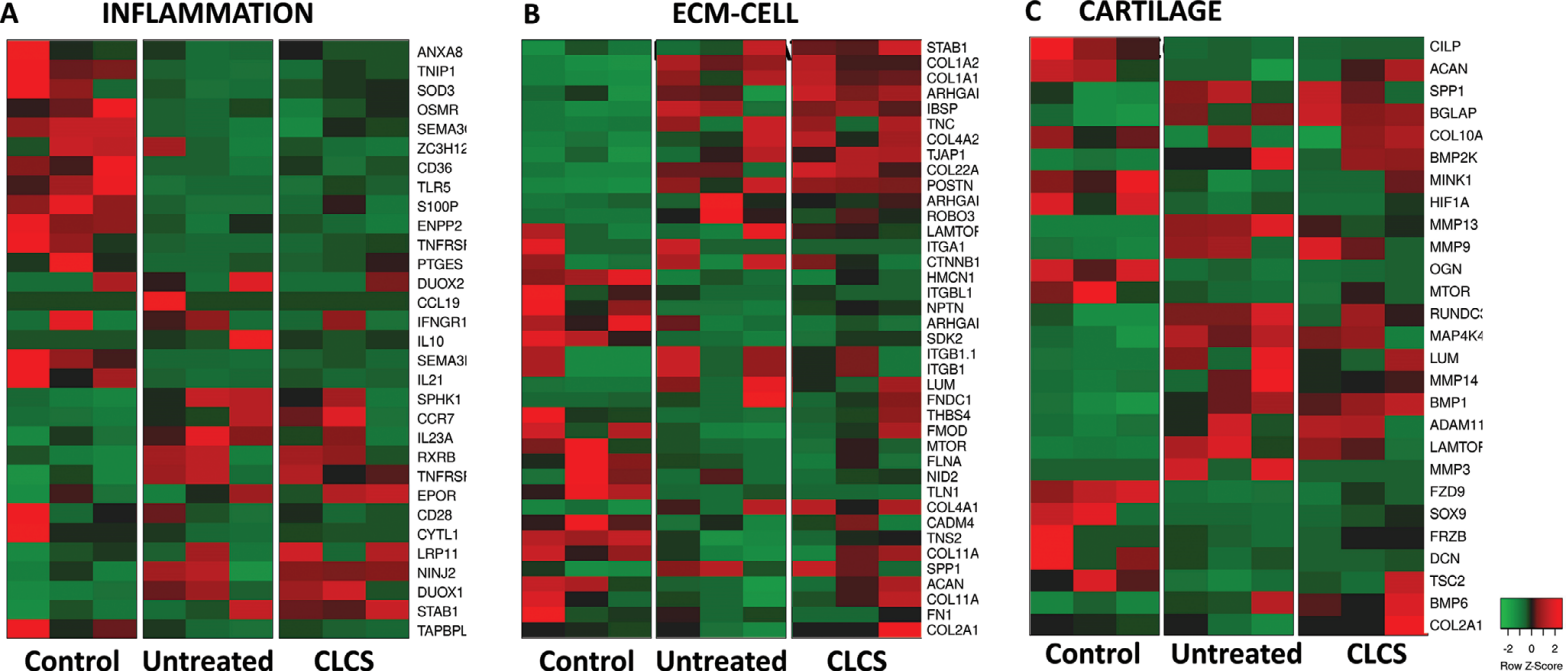
CLCS Treatment effect on Inflammation, ECM–cell communication, and cartilage homeostasis. A) Heatmap of differentially expressed genes of the inflammatory, B) ECM–cell communication, and C) cartilage homeostasis pathways between CSCL treated, untreated and healthy cartilage (control) in in vivo explants. Raw differentially expressed gene counts obtained from eXpress were mapped using heatmapper software^[115]^ and displayed as color‐coded: red represents overexpression while green underexpression.

In order to assess the organism‐specific molecular inflammatory response induced by CLCS scaffolds implanted at damaged articular cartilage, the RNA‐seq dataset was interrogated using Ingenuity Systems and Ingenuity Pathway Analysis (IPA) pathway databases. A simplified diagram of the IPA “Inflammatory pathway” displaying the differential expression of relevant inflammatory proteins and their specific receptors is shown in Figure S4 (Supporting Information). Regardless of the presence of CLCS scaffolds, cartilage injury seemed to elicit similar inflammatory‐related gene responses after injury (**Figure** **7**A). For instance, extracellular inflammatory signaling molecules IL21, SEMA3C, and TNIP1 were equally downregulated following cartilage injury both in the untreated and the CLCS samples, as were inflammatory effectors PTGES, ANXA8, and SOD3 (Figure 7A). Interestingly, adipokine receptor STAB1 was found significantly upregulated only in the presence of CLCS scaffolds (Figure 7A and Table S3, Supporting Information), suggesting modest differences between the inflammatory responses of treated and untreated rabbits.

To further explore the essential role of the molecular interaction between cells and their surrounding ECM in cartilage tissue homeostasis,^[^ ^25^ ^]^ IPA “ECM‐cell interaction” and “cartilage homeostasis” pathways were interrogated (Figures S5 and S6, Supporting Information). Similar to the inflammatory pathways, the presence of CLCS did not seem to dramatically alter the expression landscape of injury recovery (Figure 7B). Both CLCS and untreated cartilage displayed a significant increase in COL1A1, COL1A2, COL4A1 COL4A2, and COL22A1 compared to healthy cartilage (Figure 7B). However, some ECM collagenous components such as COL11A1 and COL11A2 were only found to be significantly altered in the untreated sample. Similar differences can also be observed in the expression levels of important baseline membrane glycoproteins such as ACAN or FMOD (Figure 7A), supporting the idea that CLCS helped faster extracellular reorganization postinjury, especially in their proteoglycan components. Interestingly, key regulators of cartilage homeostasis, SOX9*, MTOR*, and its indirect inhibitor *TSC2*, were only found to be significantly altered in the untreated samples. Similarly, members of the MAPK, WNT, RUNX2 and NFKB (*MAPK4K* and *MINK1, FRZB* and *FRZ9*, *RUNDC3B*, and *NFKB1* respectively), indicators of hypertrophic cartilage, were altered only in untreated samples. Overall, these results suggested a deviation from tissue regeneration pathway activation and maintenance of an active inflammatory state in untreated cartilage in comparison to CLCS samples.

**Figure 8:**
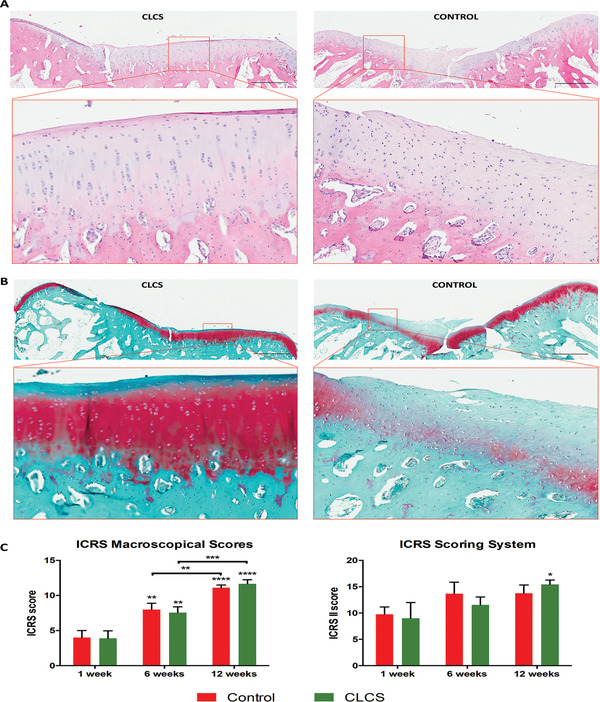
CLCS treated cartilage cellular distribution tissue structure and composition 12 weeks postsurgery. A,B) Representative images of hematoxylin and eosin staining (A) of the cellular composition and structure and safranin‐O tissue staining B) of ECM composition. All images refer to untreated empty control (CONTROL) and CLCS scaffold (CLCS) treated joint defects (and insets in red boxes) at 12 weeks post‐surgery. Scale bar represents 1 mm. C,D) ICRS macroscopic and histological scores. Results were tested for significance using nonparametric Mann–Whitney U test from the three biological replicates and two experimented observers. * was used for *p* values lower than 0.05.

CLCS treated samples macroscopical and histological analysis was performed at 12‐week postsurgery and compared to previous timepoints (**Figure** **8** and Figures S7 and S8, Supporting Information). The International Cartilage Repair Society (ICRS) I scoring system^[^ ^26^ ^]^ was utilized to evaluate the samples and compared to untreated controls (Figure 8). Macroscopically scored CLCS treated samples displayed a significant increase in scoring average both at 6 and 12 weeks in comparison to the previous timepoint (1.94 ± 0.18‐fold and 1.54 ± 0.06‐fold, *p* < 0.01 and *p* < 0.001). However, even though CLCS treated samples average score at 12 weeks post‐treatment (11.67 ± 0.47) was higher than control (11.11 ± 0.31), this difference was not statistically significant.

In addition, fixed surgical CLCS treated samples slides stained in H&E displayed nuclear and ECM staining homogeneous across the width of the trochlear groove (Figure 8A). CLCS scaffolds could not be identified anymore with the tissue showing integrity throughout the defect. CLCS treated tissue exhibited the typical columnar chondrocyte organization through the deep zone of the tissue different from the superficial zone in the higher magnification inlets. Moreover, a decrease in cellular to matrix ratio was observed when the images were compared to untreated damaged tissue at 12 weeks postsurgery (Figure 8A). In addition, untreated cartilage defects displayed a pleiotropic cellular presence and areas with increased purple nuclear staining.

Safranin O stained slides from CLCS treated samples displayed a superficial thin band of blue staining corresponding to the superficial articular cartilage zone (Figure 8B). CLCS transitional and deep zones of repaired cartilage exhibited intense red staining of the GAGs and disappeared below in the calcified and subchondral bone tissues, stained only in blue. Conversely, untreated defects displayed a heterogeneous layer of reddish Safranin O stained area across the width of the tissue with greater areas of blue staining compared healthy cartilage (Figure 8B). Moreover, untreated damaged cartilage exhibited weaker red‐stained areas in comparison to CLCS treated samples and red staining was observed in subchondral bone.

The cartilage histology slides were scored following International Cartilage Repair Society (ICRS) guidelines.^[^ ^27^ ^]^ CLCS treated samples 12‐weeks post‐treatment showed a significant increase (1.72 ± 0.08‐fold, *p* < 0.05) in average score in comparison to the first‐week post‐treatment (Figure 9D). Even though CLCS treated samples histology score averages increased over time (9 ± 2.45, 11.55 ±1.23, and 15.44 ± 0.68), untreated samples displayed nonsignificant higher score values at earlier timepoints 1 and 6 weeks (9.78 ± 1.13 and 13.67 ± 1.78) and lower score average at 12 weeks (13.77 ± 1.25) in comparison to CLCS treated samples, suggesting that the intrinsic repair mechanism of the lapine model may be not be sufficient at longer term regeneration when the CLCS scaffolds might be a better treatment option.

## 3 Discussion

Tuning the inflammatory process following injury is crucial to establish long‐term cartilage homeostasis. Unlike other tissues, articular cartilage is composed of chondrocytes and a dense ECM composed by mainly of collagen Type II and GAG. It is difficult for chondrocytes to migrate the area of injury due to the dense structure of hyaline cartilage. Moreover, due to the articular cartilage having no blood vessels, migration of cells involved in the regenerative process is more difficult than other tissues. However, by implanting a scaffold that not only recruits appropriate cells, but also tunes the inflammatory response, it is possible to increase the endogenous regenerative capacity of a tissue that is inherently difficult to regenerate. Moreover, by tuning the inflammatory response, the scaffold is able to allow for the site of injury to overcome the initial acute inflammatory phase, and redirect resources towards tissue repair stages.^[^ ^28^ ^]^

After insult, initial inflammation is normally resolved within the first week.^[^ ^29^ ^]^ In this study, dGEMRC and histology results, as well as RNA levels, suggested an enhancement of the inflammatory resolution as early as 1‐week post‐implantation. At 1‐week post‐surgery, both empty and experimental cartilage increased their ECM GAG content. It is not unusual during the first week for ECM to be produced throughout the wound healing cascade for any healing process. Starting 3 days after injury, ECM deposition is an essential step in the repair process. However, the most appropriate inflammatory response would allow for regeneration and repair without the formation of a scar.^[^ ^29^ ^]^ Due to this, ECM production becomes critical to allow for the regeneration process, while also avoiding chronic inflammation.

Therefore, further histological and molecular experiments were performed. Repression of *TNFa, NOS2, IL21, IL4, IL7* relevant proinflammatory genes, and activation of anti‐inflammatory *IL10, CXCR4* gene expression supported the enhanced inflammatory resolution. In addition, FBR absence and lack of giant body cell formation confirmed the immunomodulatory induction of the CLCS. This supporting molecular data demonstrated a mechanism for the resolution of the initial inflammation that aids to drive the regeneration process.

A differential immunomodulatory expression profile was exhibited by the CLCS treated cartilage compared to untreated empty defects. CLCS scaffolds induced a downregulation of proinflammatory cytokines^[^ ^30^, ^31^, ^32^, ^33^, ^34^ ^]^ and upregulation of anti‐inflammatory IL10 cytokine,^[^ ^35^, ^36^, ^37^ ^]^ but also activated *IL6*, *MMP3*, and *MMP13* expression as a compensatory effect for the immunomodulation.^[^ ^38^ ^]^ These results correlate with the histological differences observed in the cellular and ECM composition between both experimental groups.

Histological results 1 week posttreatment at explanted CLCS scaffolds displayed greater integration and cellular infiltration throughout the entire depth of the scaffolds. Moreover, as expected, CLCS treated samples did not demonstrate any signs of foreign body reaction.

The advantage of utilizing a biomimetic scaffold similar to the native tissue composition, as well as the scaffold having porosity that enhances cell invasion and growth favored the retention of invading cells during the initial stages of inflammation.^[^ ^4^ ^]^ The increased number of mononucleated cells in the CLCS treated defects compared to the empty scaffolds allowed for superior inflammatory resolution, observed by the lack of material encapsulation or giant body cells, as well as the molecular analysis. Both histology and molecular results suggest that CSCL is able to tune the environment where is placed, favoring a faster inflammation resolution. On the contrary, untreated samples showed thinner necrotic areas without the presence of cells or clonal activity at the apical side of the tissue, probably arriving from the bone marrow. Moreover, big rounded cells were present, which are indicative of hypertrophy and future apoptosis due to cartilage degeneration.^[^ ^39^ ^]^ This unresolved inflammatory process, further enhanced by necrosis, released additional alarms to feed back into the inflammatory process, which can easily lead to chronic inflammation and cartilage disease. Likewise, Safranin O staining was chosen to assess the GAG content produced during the first stages of the cartilage repair process. Bluish‐green fast green staining of collagen from the scaffold further confirmed the presence and integration of the scaffolds within the native tissue. Cartilage matrix deposition and the presence of GAGs were still reduced at this stage. Conversely, empty defects showed some staining at the bottom, displaying some hypertrophic round chondrocytes under a layer of acellular collagenous bluish stained matrix, which is indicative of a scar formation process.^[^ ^40^ ^]^ These results, not observed in CLCS treated cartilage, suggest that CLCS scaffolds induced the repression of scar tissue formation. Further development of the scar in the articular cartilage defect normally evolves into degenerated cartilage or fibrocartilage with limited functional capacity and probably results in further tissue damage, chronic inflammation, and disease.^[^ ^39^ ^]^

Cytof analysis showed a snapshot of the infiltrating inflammatory cells at the site of implant at the early time point (1 week postimplant). Instead of evaluating specific staining, we focused our attention to identify the overall immune response at the site of implant. It has been recently reported that type 2 immunity exhibits many host‐protective functions, including maintenance of metabolic homeostasis, suppression of excessive type 1 inflammation, maintenance of barrier defense, and regulation of tissue regeneration.^[^ ^41^ ^]^ This type of immunity has been described in multiple different tissues and animal models.^[^ ^42^ ^]^ The presence of significantly higher infiltration of CD^3+^ together with CD^4+^ (not significantly higher, but in some samples was found—note outliners in Figure 4B) suggests the possibility of activation of type 2 immunity induced by CSCL. Moreover, as recently reported, biological scaffolds depend on their physicochemical features to promote the polarization of infiltrating macrophages towards the alternative M2 phenotype.^[^ ^28^ ^]^ M2 macrophages provide key growth factors, which aid in wound repair and the promotion of fibrogenesis. Furthermore, these cells can recruit TH2 effector cells, which further polarize the response. We found a significant difference in both CD^11b+^ and CD^206+^ cluster (M2 marker), suggesting the high presence of these specific cell types at the site of implant further confirmed by the overexpression of IL‐10 identified by RNA array and RT‐PCR. In fact, M2 macrophages can dampen the inflammatory response by releasing IL‐10, which can promote regulatory T cell differentiation. Overall, the type 2 response is complex, targeting many cell types and having both beneficial and pathogenic features that are not mutually exclusive. Moreover, we had several limitations of antibody cross‐reactivity due to the lapine model. However, we were able to confirm this unique immune environment and evaluated how this early response could be translated to a later time point in terms of tissue homeostasis.

Long‐term cartilage tissue regeneration was evaluated 12 weeks postsurgery.^[^ ^43^ ^]^ Even though complete regeneration was not achieved at 12 weeks, the results point towards an enhanced tissue repair process, with cellular and molecular levels indicating this at the mid‐timepoint. Histological and RNA expression outcomes suggest that the immunomodulatory effect of CLCS treated insults influenced the repair process and following 12‐weeks of tissue repair, and molecular tools further confirmed the remodeling of the tissue and reduction of the inflammatory environment.

Transcriptome analysis allowed for the identification of molecular pathways differentially activated or repressed in response to immunomodulatory CLCS scaffold treated cartilage compared to untreated cartilage defects.^[^ ^44^, ^45^ ^]^ Compared to previous work,^[^ ^46^ ^]^ differential expression of several members of the TNFa family *TNIP1, TNFRSF1A* and *TNFRSF11B*, *NFkB1*, *MMP9* and *MMP13* and interleukins *IL21* and *IL23A* in comparison to healthy controls was observed.

PCA analysis of the RNA expression data clearly demonstrated sample replicates in three distinct clusters. Healthy control cartilage RNA data clustered at one end of the bidimensional representation of the expression, while untreated empty defects RNA expression data clustered at the opposite side, suggesting that the homeostatic disruption produced with the insult at surgery was not restored by intrinsic mechanisms at 12‐weeks postsurgery. Furthermore, the existing differences in gross RNA expression from CLCS treated cartilage to healthy controls suggest a slower degree of tissue maturation. Conversely, an intermediary state of pathway activation is required to regain tissue homeostasis.

Unique signature profiles between untreated and CLCS treated cartilage was analyzed. As expected, untreated cartilage defects displayed a significant downregulation in cartilage‐specific ECM composition and maturation genes such as *ACAN*, *COL11A1*, *COL11A2*, *SOX9*.^[^ ^47^ ^]^ Moreover, inhibition of *MTOR* expression, described as a prior step to OA development in a rat model and associated with OA in humans,^[^ ^48^, ^49^, ^50^ ^]^ was found among the unique repressed genes in untreated empty defect samples, further confirming a damaged cartilage tissue state. Additionally, unique expression of biomimetic CLCS scaffolds treated cartilage defects included several downregulated proinflammatory genes such as *CD28, ENPP2, and OSMR*, coding for protein activators of the T‐cell inflammatory response,^[^ ^51^ ^]^ and inflammatory‐related cartilage degenerative conditions.^[^ ^52^, ^53^, ^54^ ^]^ IPA was utilized to determine significant cartilage regulatory pathways within the data sets and to discover any potential regulatory networks and causal relationships between biomimetic CLCS treated cartilage immunomodulation and regeneration at 12‐weeks postsurgery.

Transcriptome profiling suggests that the immune environment is being modulated favorably towards a repair process in CLCS implanted cartilage samples 12 weeks postsurgery. CLCS scaffold immunomodulatory induction could be observed when the expression profiles were compared to untreated cartilage defects. Untreated cartilage displayed a significant increase in pro‐inflammatory *IL23* expression observed as early as 1‐week post‐surgery. In addition, untreated cartilage defects exhibited a significant decrease in *PTGES, TNIP1, IL21*, semaphorins and annexins RNA expression coding for necessary anti‐inflammatory molecules related to TNFa receptors and NF‐kB protein complex,^[^ ^55^ ^]^ activation of inflammatory condition through T cells,^[^ ^56^, ^57^ ^]^ chondrocyte inflammation and osteogenic differentiation.^[^ ^58^, ^59^ ^]^ The statistically significant altered expression in untreated cartilage defects indicates the presence of a proinflammatory environment within the tissue and lack of anti‐inflammatory capacity to counteract it. Conversely, CLCS treated cartilage defects did not exhibit significant alterations in the same mRNA molecules, or they were modulated and less significant in comparison to the untreated defects. These results suggest that CLCS scaffolds implanted after cartilage insult, induced the repression of proinflammatory signals. Many times this initial inflammatory process is not well resolved, and the organism may enter into a continuous state of inflammation becoming a chronic condition and resulting in immature fibrotic cartilage tissue.^[^ ^60^, ^61^ ^]^ However, this chronic inflammatory process can easily damage the articular cartilage tissue and any TE attempt to regenerate the tissue must first suppress proinflammatory processes in order to produce an environment that facilitates mature and functional cartilage tissue growth.^[^ ^62^ ^]^

The ECM plays a tremendous instructive role, containing cytokines, GFs, and important protein and GAG epitopes that are recognized by chondrocytes. Upon receiving various signals, chondrocytes respond accordingly resulting in cellular protein secretion, proliferation, or cell death which affects tissue dynamics during development, as well as healthy mature function, repair and disease stages.^[^ ^63^ ^]^ There were distinct differences in RNA expression of ECM–cell interaction components between CLCS and untreated cartilage defects.

Untreated cartilage defects displayed an aberrant of ECM GAGs and glycoproteins encoding mRNA expression in comparison to healthy cartilage. Abnormal expression of important ECM members such as ACAN, DCN, LUM, FMOD, and NID2 have a direct effect on cartilage composition, transmitted to chondrocytes through membrane receptors further signaling through SOX9 or MAPK, RUN, WNT, and MTOR signaling pathways to counteract ECM limitations with increased turnover and further degradation of the cartilage.^[^ ^64^, ^65^, ^66^ ^]^

CLCS scaffold treated cartilage also showed identical expression of cell adhesion molecules *CADM4, SDK2*, and *NPTN* RNA compared to the healthy control samples, which are all proteins essential for the selectivity of cellular response to the environment.^[^ ^67^, ^68^, ^69^, ^70^, ^71^, ^72^ ^]^ These results suggest that CLCS treated cartilage affected the ECM composition by inducing a similar expression of transmembrane receptors to healthy cartilage, increasing the chances for restored homeostasis after tissue repair. Integrins, through ligand binding and signal transduction activation, play an essential role in the cellular interaction with the surrounding ECM.^[^ ^60^, ^73^ ^]^ Untreated cartilage defects displayed a significant decrease in *TNS2* and *ITGBL1* RNA expression, whereas biomimetic CLCS scaffold treated cartilage did not show significant differences in *TNS2* expression, even reducing *ITGBL1* expression, suggesting CLCS scaffolds altered cellular interaction with the surrounding ECM.^[^ ^74^ ^]^

Differential gene expression in *BMP1, BMP2*, and *BMP2K* activating the TGFb family and SMAD signaling,^[^ ^75^ ^]^ *FRZB* and *FRZ9*, related to WNT,^[^ ^76^ ^]^ *LAMTOR1* and MTOR inhibitor coding gene *TSC2*,^[^ ^77^ ^]^ *NF‐kB1*,^[^ ^78^ ^]^ and *MAPK4K* and *MINK1* from the MAPK^[^ ^79^ ^]^ indicated a differential induction of these inflammatory signaling pathways in response to CLCS implants in comparison to untreated damaged cartilage. In addition, differential mRNA expression in *COL4* and *COL11*, *ACAN* and *MMP13* was observed further confirming the scaffolds induction of genes that are involved in cartilage homeostasis and OA.^[^ ^80^ ^]^ Moreover, untreated empty defects showed increased downregulation in *DCN* mRNA expression compared to CLCS treated cartilage. DCN, a member of the SLRPs, interacts with collagen to organize the structure of ECM, limits cellular access to proteinases, GFs and cytokines,^[^ ^81^ ^]^ and inhibits of the TGFb family and SMAD inflammatory signaling pathway, allowing for better wound healing and regeneration.^[^ ^82^ ^]^

*MAPK4K* and *MINK1* expression alterations in untreated cartilage defects modulated with biomimetic CLCS scaffold implants were also revealed. This was confirmed by the dysregulation of MAPK signaling, a major component of the immune reaction, inflammatory condition, and cartilage degeneration.^[^ ^83^, ^84^, ^85^, ^86^ ^]^ Excitingly, no significant differences were observed between CLCS treated defects and healthy cartilage explants in the same MAPK enzymes, reinforcing the scaffolds immunomodulatory effects at 12 weeks postsurgery.

Untreated damaged cartilage samples displayed a significant reduction in *SOX9* RNA expression, encoding for the cartilage master regulator protein, possibly linked to SMAD, MAPK/NF‐kB, MTOR, and WNT signaling dysregulation observed in the same untreated defects. SOX9 dysregulation has catastrophic effects on cartilage ECM composition, and the negative feedback loop leads to cartilage disease.^[^ ^87^, ^88^ ^]^ CLCS treated cartilage did not show any significant differences in *SOX9* expression in comparison to healthy cartilage, strongly suggesting a beneficial regenerative outcome induced by early immune modulation. Importantly, CLCS treated cartilage displayed a significant reduction in *LPR1* mRNA expression indicating a direct relation to MMP regulation via endocytosis.^[^ ^89^ ^]^ As a possible consequence of *LPR1* mRNA repression, treated cartilage exhibited a significant increase in *MMP9* and *MMP13* RNA expression, suggesting that CLCS treated cartilage was still under ECM remodeling, but not yet in homeostasis compared to healthy cartilage tissue.

The level of regeneration produced over the 12 weeks after CLCS scaffold implantation was followed via histological and molecular studies. Overall, a better level of regeneration was achieved in the CLCS treated cartilage experimental group, displaying tissue organization and composition similar to healthy tissue, and accompanied by a differential pattern of expression compared to untreated cartilage, which showed clear histological and molecular signs of an ongoing inflammatory expression process. Although, gross images exhibited a continuous surface in both experimental groups, rough and irregular areas could be observed on the surface of untreated cartilage, suggesting that the tissue integrity was not sufficient and probably not responding properly to the physical demands of the tissue. Gross appearance is often used to grade the quality of the cartilage and to assess the level of degeneration whenever invasive techniques with better resolution are available.^[^ ^90^ ^]^

CLCS treated cartilage appeared to have a better continuity on the surface whereas untreated samples exhibited empty areas and discontinuous superficial cartilage tissue. In addition, CLCS treated cartilage displayed a homogenous depth similar to healthy cartilage that was maintained throughout the entire width of the insulted area. Conversely, empty defects displayed thicker areas where bone marrow invasion could be observed from the subchondral bone and thinner cartilage areas. Differential cellular composition and organization could also be observed between both groups through the characteristic superficial, middle, deep and calcified zones. CLCS treated samples displayed flat cellular bodies, which were observed at the surface of the cartilage, while rounder chondrocytes were found in deeper zones. Moreover, the typical columnar organization of the cells could be recognized in deeper areas. At high resolution, CLCS treated cartilage images resembled healthy cartilage appearance. This was not present in untreated cartilage. The cellular composition was abnormal, without any morphological or size differences between zones displaying an anisotropic organization. Hunziker et al. described a similar organization in immature rabbit cartilage.^[^ ^90^ ^]^ Therefore, it could be inferred that the biomimetic CLCS scaffolds contributed to a better reorganization of cells and faster maturation into cartilage tissue that appears similar to naive healthy tissue.

Regenerated cartilage GAG composition was assessed using the Safranin O staining. Healthy cartilage is characterized by increased GAGs content in comparison to other tissues. GAGs such as ACAN, CS, HS, and DS provide structural and biological functions required by a very specific and demanding articular cartilage tissue.^[^ ^78^ ^]^ CLCS treated cartilage exhibited a greater and intense brown band indicative of important GAG quantities in the regenerated tissue. The superficial zone displayed homogeneous thin band with little or no staining. This is consistent with mature articular cartilage composition, where GAG content is less abundant in the superficial lamina splendens and increases throughout the depth of the articular cartilage.^[^ ^91^ ^]^ Taken together, the histological results showed CLCS treated cartilage defects to be in a superior regenerative stage at 12 weeks postsurgery. Both the cellular and ECM components appearance was closer to healthy cartilage than to untreated cartilage. Biomimetic CLCS scaffolds induced an immunomodulatory organism reaction during the initial phase of repair at the tissue, cellular and molecular levels that beneficially affected the repair process during the first 12 weeks of regeneration. However, molecular mechanisms underlying cartilage homeostasis and matrix production were analyzed to confirm the higher quality of the CLCS induced new cartilage. Not only did the CLCS treated cartilage display superior properties observed in histology, but the RNA expression processes ongoing at 12 weeks were better suited for the maintenance of the tissue homeostasis and long‐term regeneration.

The results observed demonstrated physical support and integration into the tissue allowing for cellular infiltration, tissue remodeling and regeneration, while also modulating the early phase inflammatory environment. Excitingly, biomimetic porous collagen scaffolds functionalized with CS exhibited increased modulatory capacity against the chronic inflammation produced after cartilage damage and modulated the inflammatory response towards an environment of regeneration rather than inflammation in comparison to untreated controls. This study therefore demonstrates the utility of biomimetic immunomodulatory 3D scaffolds for cartilage tissue regeneration in vivo and provides a platform for further preclinical studies; increasing the study sample size and utilizing larger mammalian models. Moreover, this study was performed in a lapine model, and it is well established that this species has superior cartilage repair properties when compared to larger animal models (including humans). Therefore, the results of this study must be confirmed in a model that has cartilage repair and regeneration abilities more similar to that of humans prior to translating the use of the CLCS scaffold in the clinic.

## 4 Conclusion

The capacity of biomimetic modifications to collagen scaffolds in the form of CS to modulate the immediate immune environment and support tissue integration results in increased tissue regeneration, which was tested in vivo in an orthotopic lapine model. The immunomodulatory properties of functionalized CLCS scaffolds in vivo were tested at 1 week postsurgery via a comprehensive molecular analysis, as well as through routine histological and noninvasive cartilage assessment procedures. A whole transcriptome analysis allowed for the understanding of the molecular pathways induced by the CLCS treated cartilage beneficial for the tissue regeneration after injury and the reduction of the accompanying chronic inflammatory process leading to disease states. Last, the porous biomimetic CLCS scaffolds showed improved histological molecular and cellular composition and organized tissue structure in comparison to untreated controls at 6 and 12 weeks postsurgery. The results presented suggest CLCS acellular scaffolds can be great candidates for cartilage tissue repair applications due to their ability to modulate the immune environment in favor of a regenerative process.

## 5 Experimental Section

### Porous Collagen‐Chondroitin Sulfate Scaffold Fabrication

CLCS functionalized scaffolds were fabricated from bovine tendon extracted type I Collagen using a freeze‐drying method.^[^ ^14^ ^]^ 200 g of purchased collagen type I in acetic acid (5% w/v; Nitta Casings Inc., NJ, USA) were dissolved in 1 L deionized water at a final concentration of 10 mg mL^‐1^ and mixed thoroughly. The collagen suspension was then precipitated by the addition of 0.1 m sodium hydroxide solution until the pH reached 5.5. CS (Carbosynth, Berkshire, UK) at a weight molar ratio 10:1 (Collagen:CS) was added to the solution and thoroughly mixed. 81 µL of 1.5 × 10^‐3^ m BDDGE were finally added to allow 24 h crosslinking at RT plus another 24 h at 4 °C. After water rinsing, the final slurry was poured onto a 24‐well culture plate and freeze‐dried until the resulting porous scaffolds were formed. The scaffolds were sterilized by UV irradiation for 4 h under a laminar flow hood.

### Scanning Electron Microscopy (SEM)

The morphology of the CLCS scaffold was characterized by scanning electron microscopy (SEM). Scaffolds were coated by 7 nm of Pt/Pl for scanning electron microscope (SEM; Nova NanoSEM 230, FEI, Hillsboro, OR, http://www.fei.com) examination.

### Compression Testing

CSCL scaffolds of 0.5 cm thickness were soaked in PBS and loaded on UniVert Mechanical Test System. A Load Cell of 10 N was calibrated and used to perform a compression test with a stretch magnitude of 35% and a stretch duration of 60 s and a relaxation time of 60 s. A minimum of three replicates were performed and recorded for each condition.

### Rheology

Wet scaffolds of 1 mm thickness and 8mm diameter were analyzed using an Anton Paar/MRC 302 rheometer equipped with an aluminum 8 mm insert plate. Both empty scaffolds and cellularized scaffolds collected at day 7 were characterized. An amplitude sweep test (log ramp 0.001%/10%, angular frequency of 10Hz, 25 recorded points, *T* of 37 °C) was used to verify the range of linear viscoelasticity. Frequency response was measured by frequency sweep tests in the range 1000/0.1 rad s^‐1^ (shear strain of 0.1%, 40 data point, *T* of 37 °C). Storage modulus and loss moduli measures were reported as an average of three samples collected at 1 rad s^‐1^ angular frequency.

### Fourier Transform Infrared Spectroscopy (FTIR)

The samples were analyzed in transmission mode at resolution 4, 64 points, over the range of 500–4000 cm^−1^ using a Nicolet 6700 spectrometer (Thermo‐Fisher Scientific, Waltham, MA, http://www.thermofisher.com). The FTIR spectra were reported after background subtraction, baseline correction, and reported on the graph after background subtraction, baseline correction, and normalization on Amide I, within the range of 500–1800 cm^−1^.

### In Vivo Articular Cartilage Rabbit Orthotopic Model

A total of twelve 4 to 5 month old New Zealand white (NZW) rabbits weighting 2 to 3 kg were selected providing 24 knees for the experimental groups. NZW underwent a surgical procedure to generate a critical size defect at the trochlear groove of the knee joint.^[^ ^92^ ^]^ All procedures followed the IACUC protocol (IS00004559) established by Houston Methodist Research Institute's Institutional Animal Care and Use Committee in accordance with the guidelines of the Animal Welfare Act and the Guide for the Care and Use of Laboratory Animals. Total anesthesia was induced by the CMP staff using 1–5 mg kg^‐1^ benzodiazepine intramuscularly and maintained with the continuous administration of inhaled anesthetic isoflurane gas (1–5%). Following appropriate aseptic procedures, the lateral aspect of the leg was prepped.

First, a longitudinal skin incision was made directly over the bilateral knee joints. Arthrotomy was performed via standard medial parapatellar dissection and the capsule was incised along the medial border of the quadriceps tendon, patella, and patellar tendon, taking care not to violate the extensor mechanism. Medial soft tissue release, lateral luxation of the patella, and hyperflexion of the knee were then performed to expose the weight‐bearing surface of the femoral condyles. A full‐thickness osteochondral defect, 4 to 6 mm in diameter by 0.5 to 1 mm in depth, was created in the trochlear groove using a clinical‐grade orthopedic microdrill. Both left and right trochleae were insulted, and the use of continuous saline irrigation helped to remove debris and to reduce heat necrosis to chondrocytes. A 5 mm diameter by 1 mm height CLCS scaffold was implanted at the right leg defect, while the left knee was left untreated and used as a control defect. Patellar reduction was followed by fascial and skin closures performed with 2‐0 Vicryl and 4‐0 Monocryl (Ethicon, NJ, USA) absorbable sutures respectively.

CMP staff performed the postoperatory care in individual cages of the animals under CMP standard operating procedures. Analgesics and antibiotics were administered if indicated by CMP staff, avoiding the use of carprofen as pain medication due to musculoskeletal healing interferences.^[^ ^93^ ^]^ At the end of the experiments, the animals were humanely euthanized by the non‐inhalant pharmaceutical method, consisting of 0.22 mg kg^‐1^ administered through the intravenous route.

### Delayed Gadolinium‐Enhanced Magnetic Resonance Imaging

All rabbits were transported under total anesthesia and monitored for immediate postsurgery (1 week and 12 weeks) dGEMRIC to assess the adequacy of the tissue deposition and the assessment of repair morphology process.^[^ ^94^ ^]^ Omniscan (gadodiamide; GE Healthcare, IL, USA) contrast agent at 0.2 mL kg^‐1^ (0.1 mmol kg^‐1^) was intramuscularly administered 10 min prior to the imaging procedure. Conventional coronal, radial, and sagittal MRI imaging at 3 Tesla whole‐body MRI scanner (Siemens MAGNETOM Vida, Siemens Healthineers, Germany) was performed using a 16‐channel transmit‐receive knee coil (Philipps Medical Systems AG, The Netherlands). A standard protocol for dGEMRIC T1 mapping acquisition was followed and data were processed by the core staff. The same protocol was followed in both experimental and control knees, including localizers, as follows. Coronal imaging, 3D T1‐weighted turbo field echo sequence for dGEMRIC (repetition time/echo time 4.6/1.59 ms, five inversion delays 200‐/300‐/400‐/800‐/1200 ms, flip angle 15°, matrix 260 × 198, field of view 13 cm, slice thickness 1.5 mm, bandwidth 478.9 Hz/Px, acquisition time 23:50 min, 79 slices). Radial imaging, 2D multiecho spin‐echo sequences for T2 mapping of cartilage (repetition time 2000 ms, six echo times 13‐/26‐/39‐/52‐/65‐/78 ms, flip angle 90°, matrix 240 × 194, field of view 10 cm, slice thickness 2 mm, bandwidth 291 Hz/Px, acquisition time 16:32 minutes, 30 slices); and a morphologic radial, 2D proton density‐weighted turbo spin‐echo sequence without fat suppression (repetition time/echo time 3085/30 ms, flip angle 90°, matrix 304 × 299, field of view 7 cm, slice thickness 2 mm, bandwidth 198.6 Hz/Px, acquisition time 9:25 min, 30 slices).

### Ex Vivo Histological Staining

At the end of in vivo experiments, tissue of interest containing the implanted scaffolds was harvested and fixed in 10% NBF overnight. Samples were then transferred to the CMP pathology department at the HMRI for wax embedding and tissue sectioning. Embedded samples were sectioned to 4 mm using a microtome, flattened in a 45 °C water bath and immobilized into histology slides. Before staining, the sections were deparaffinized and hydrated through a series of successive 10 min incubations. H&E staining following standard staining protocol using Wiegert's Iron Hematoxylin solution and 1% Eosin Y (Sigma‐Aldrich, MI, USA) was performed. For Safranin O staining, 0.02% fast green (Sigma‐Aldrich, MI, USA) and 1% Safranin O (Sigma‐Aldrich, MI, USA) were used. After each specific staining protocol, the samples underwent a dehydration process with 95%, 100% ethanol, and Xylenes. Finally, mounted glass cover slides (Corning, NY, USA) were imaged using a an automated Aperio Scanscope AT Turbo Scanner (Leica Biosystems, IL, USA) with the supporting Aperio ImageScope (Leica Biosystems, IL, USA) pathology slide viewing software.

### Imaging Mass Cytometry/CyTOF Antibody Staining

Metal‐labeled antibodies were prepared according to the Fluidigm protocol.^[^ ^95^ ^]^ Antibodies were obtained in carrier/protein‐free buffer and prepared using the MaxPar antibody conjugation kit (Fluidigm). After determining the percent yield by absorbance measurement at 280 nm, the metal‐labeled antibodies were diluted in Candor PBS Antibody Stabilization solution (Candor Bioscience) for long‐term storage at 4 °C. Antibodies used in this study are listed in Figure 4.

Samples were baked at 60 °C overnight, then dewaxed in xylene and rehydrated in a graded series of alcohol (ethanol absolute, ethanol:deionized water 90:10, 80:20, 70:30, 50:50, 0:100; 10 min each) for imaging mass cytometry. Heat‐induced epitope retrieval was conducted in a water bath at 95 °C in Tris buffer with Tween 20 at pH 9 for 20 min. After immediate cooling for 20 min, the sections were blocked with 3% bovine serum albumin in tris‐buffered saline (TBS) for 1 h. For staining, the sections were incubated overnight at 4 °C with an antibody master mix (Figure 3E). Samples were washed four times with TBS/0.1% Tween20. For nuclear staining, the sections were stained with Cell‐ID Intercalator (Fluidigm) for 5 min and washed twice with TBS/0.1% Tween20. Slides were air‐dried and stored at 4 °C for ablation. The sections were ablated with Hyperion (Fluidigm) for data acquisition. Imaging mass cytometry data were segmented by ilastik and CellProfiler. Histology topography cytometry analysis toolbox (HistoCAT) and R scripts were used to quantify cell number, generate tSNE plots, and perform neighborhood analysis. For all samples, cellular densities were averaged across three images per specimen.

### Tissue Repair Evaluation

The macroscopic assessment of the repaired tissue was evaluated using the ICRS I macroscopic scoring system, which considers the degree of defect repair, the integration to the border zone, and the macroscopic appearance.^[^ ^26^ ^]^ Two external observers, both blind to the treatment, independently scored the specimens.

The histological experimental samples were analyzed at the optical microscope to evaluate the histological parameters established by the ICRS; each criterion was evaluated based on the visual analog scale and graded from 0 to 3.

### Gene Expression Analysis

RNA was extracted from snap‐frozen harvested cartilage tissue samples previously stored at ‐80 °C. First, a 30 s homogenization was performed with a PowerGen 125 tissue homogenizer (ThermoFisher Scientific, MA, USA) in 1 mL of Trizol (Life Technologies, ThermoFisher Scientific, MA, USA). After homogenization, the samples were centrifuged at 12 000 g for 10 min at 4 °C and the supernatant was collected into a new 1.5 mL tube. The samples were thoroughly mixed with 100 mL chloroform (Sigma‐Aldrich, MI, USA) and incubated at RT for 2 min. A centrifugation cycle for 15 min at 12 000 g and 4 °C was performed to separate the RNA aqueous phase. RNeasy mini kit (Qiagen, MD, USA) and its total RNA isolation protocol was applied following manufacturer's indications. On‐column DNA digestion treatment with DNAse (Sigma‐Aldrich, MI, USA). As a final step, the RNA was eluted in 30 µL of RNase‐free water and quantified using a ND1000 spectrophotometer (NanoDrop, ThermoFisher Scientific, MA, USA). The cDNA was synthesized using iScript cDNA synthesis kit (Bio‐Rad, CA, USA) from 0.5 mg total RNA at 25 ng µL^‐1^ concentration in the RNA reverse transcription master mix. A RNAse‐free water (Qiagen, MD, USA) without RNA sample was included as a negative control to confirm retro‐transcription experimental success. The final mixture was loaded into a CFX96 RT‐PCR detection system thermal cycler machine (Bio‐Rad, CA, USA). StepOne real‐time PCR system (Applied Biosystems, ThermoFisher Scientific, MA, USA) with SYBR green method was used to amplify a specific amount of cDNA from a target gene that could be detected and quantified using specific TaqMan target probes (Applied Biosystems, ThermoFisher Scientific MA, USA). Nonreverse transcribed RNA, RNase‐free water, and reverse transcribed RNase‐free water were used as negative controls to confirm the success of the whole experimental process.^[^ ^96^ ^]^ The rabbit specific TaqMan target probes used were: TNFa (Oc03397715_m1), NOS2 (Oc03398289_m1), IL4 (Oc04096359_m1), IL1B (Oc03823250_s1), ADAMTS5 (Oc03395870_m1), MMP13 (Oc03396899_m1), IL10 (Oc03396940_m1), MMP3 (Oc03397816_m1), IL6 (Oc03822686_s1), ACTB (Oc03824857_g1) and GAPDH (Oc03823402_g1)

### Inflammatory PCR Array

Rabbit explants RNA extracted 1 week postsurgery were assessed using Rabbit Inflammatory Chemokines & Receptors RT2 Profiler PCR Array (Qiagen, MD, USA). cDNA synthesis was performed using the RT2 First Strand Kit (Qiagen, MD, USA) from 0.5 µg total RNA. Before cDNA conversion, genomic elimination buffer (Qiagen, MD, USA) was used to ensure genomic DNA elimination. Reverse transcription master mix was made adding RT2 First Strand Kit master mix into the thermal cycler machine, programmed to perform the cDNA conversion reaction. RT2 SYBR Green RT2 qPCR master mix (Qiagen, MD, USA) was added to the samples and loaded onto the array plates. ABI 7500 Fast Sequence Detection System (Applied Biosystems, ThermoFisher Scientific, MA, USA) was programmed relative expression was determined using data from the real‐time cycler and the ∆∆CT method. The resulting Ct values were analyzed through the SABiosciences Web‐based PCR Array Data Analysis Software version 3.5.

### RNA Sequencing

Extracted RNA from explanted rabbit articular cartilage tissue was totally sequenced and analyzed for in depth molecular understanding. Only high‐quality isolated RNA samples were used in the downstream analysis, checked using the Agilent 2100 bioanalyzer (Agilent Inc, CA, USA). High‐quality total RNA was then processed for sequencing library preparation through mRNA selection (polyA tail mediated) and fragmentation (TruSeq kit protocol). These fragmented mRNA molecules were converted to cDNA libraries with TruSeq adaptor sequences in order to allow multiple libraries to be run on a single sequencing lane. For this experiment, the sequencing run was deemed successful after the proprietary quality controls were passed.^[^ ^97^ ^]^ Base sequence quality scores (Q scores) threshold was set above Q30. FastQC was used to plot short k‐length nucleotides (k‐mer) or single sequences overrepresented at certain positions in the reads indicating issues at library preparation or library contamination.^[^ ^98^ ^]^ All library preparation and sequencing processing was performed at the Epigenomics Core of Weill Cornell Medical College (New York, USA).

To analyze RNA sequencing data, an in‐house bioinformatics pipeline was used based on previously published works.^[^ ^99^ ^]^ A schematic of the analysis pipeline is provided in Figure 5A. FastQ files that contained high‐quality RNA sequencing reads were aligned to the “oryCun2.0.”^[^ ^100^ ^]^ In this process, “Tophat” aligned reads to “oryCun2.0” using the short‐read aligner “Bowtie” prior to identification of splice junctions that lie between exon regions. Following alignment, “Tophat” produced the required sequence alignment map (SAM) files required for further analysis such as, alignment viewing or the identification of differentially expressed genes.^[^ ^101^ ^]^ Following alignment to the reference genome, SAM or the binary version BAM output files were analyzed using “eXpress” software package to guide downstream specific analysis tasks such as PCA and identification of differentially expressed genes. The gene count matrix was used to filter transcripts that were present or absent in each of the experimental conditions; the lists of genes were compared using Venn diagrams via the InteractiVenn platform.^[^ ^102^ ^]^

DEseq2 was then used to calculate differential gene expression between sample types. In this package for “R” programming environment, the number of reads per sample was first normalized using an internal negative binomial model.^[^ ^103^ ^]^ The significance level of FDR adjusted *p*‐value of 0.05 was used to identify differentially expressed genes. Raw and processed RNA‐Seq data are deposited in the Gene Expression Omnibus database with accession number GSE149098.

Finally, the platforms PANTHER and WebGestalt were used to perform statistical overrepresentation/enrichment tests.^[^ ^104^, ^105^ ^]^ All annotation sets were tested for overrepresentation (Binomial test, FDR < 0.05) comparing the lists of genes expressed in each experimental condition to those expressed by a standard genome of reference. Similarly, all annotation sets were tested for statistical enrichment using the lists of differentially expressed genes in each experimental condition (FDR <0.05). The results of statistical enrichment testing are displayed showing the differential distribution of significantly enriched clusters of genes compared to the overall expression tendency within samples. IPA software was used for the interpretation and analysis of the data and elucidate biological interdependencies between differentially expressed genes of the inflammatory, ECM–cell and cartilage homeostasis customized pathways.^[^ ^106^ ^]^ Heatmapper software^[^ ^107^ ^]^ was used to display the results as color‐coded.

### Statistical Analysis

All experimental data distributions were assessed for normality using the Kolmogorov Smirnov test. For data that did not follow a normal distribution, a non‐parametric Kruskal–Wallis test was applied to determine significant differences in the median between groups. A Mann‐Whitney U test was then applied ad post hoc to determine significant differences between specific group pairs for median scores (IHC) between, controls versus study groups. Data with a normal distribution, one‐way and two‐way Analysis of Variance (ANOVA) tests (GraphPad Prism 6, CA, USA) followed by Dunnett's, Tukey ´s, or Sidak's post hoc test, were used to determine significant differences between groups. The test statistic and corresponding *p* value were reported, and statistical significance was defined as *p* < 0.05. All statistical analysis for each data set described is listed in the figure legends.

## Conflict of Interest

The authors declare no conflict of interest.

## Author Contributions

G.B.M. and F.T. involved at all levels from study conception, experimentation, data collection/analysis assisted by A.S. and F.B.N. M.H., S.S., and F.C. performed the surgeries and assisted with MRI protocol and interpretation supervised by P.M. M.Q.; RNAseq data analysis. P.R.; Multivariate statistics. T.J./E.C.; macro and pathology scoring and collation. E.T., L.W.F., P.M., F.T., A.B., and F.P. study management, data analysis, interpretation. All authors reviewed and edited the final version of the manuscript.

## Supporting information

Supporting Information

## Data Availability

Research data are not shared.
