# Supplementary material for: Biomimetic Scaffolds Modulate the Posttraumatic Inflammatory Response in Articular Cartilage Contributing to Enhanced Neoformation of Cartilaginous Tissue In Vivo
Source: Adv Healthc Mater. 2021 Oct 28;11(1):2101127. doi: 10.1002/adhm.202101127 (PMC11469755; doi:10.1002/adhm.202101127)
Supplement: Supplementary file 1 — Supporting Information [file ADHM-11-2101127-s001.pdf]

© 2021 Wiley-VCH GmbH

ADVANCED HEALTHCARE MATERIALS

Supporting Information

for *Adv. Healthcare Mater.*, DOI: 10.1002/adhm.202101127

Biomimetic scaffolds modulate the post-traumatic inflammatory response in articular cartilage contributing to enhanced neo-formation of cartilaginous tissue in vivo

Guillermo Bauza-*Mayol*[1,2,3]*, Marcos Quintela*[3]*, Ava Brozovich*[1,2,4]*, Michael Hopson*[2]*, Shazad Shaikh*[2]*, Fernando Cabrera*[1,2]*, Aaron Shi*[1,2]*, Federica Banche Niclot*[1,6]*, Francesca Paradiso*[1,2,3]*, Emma Combellak*[5]*, Tom Jovic*[5]*, Paul Rees*[2]*, Ennio Tasciotti*[1,2,7]*, Lewis W Francis*[1]**, Patrick Mcculloch*[2]*, Francesca Taraballi*[1,2]***

Biomimetic scaffolds modulate the post-traumatic inflammatory response in articular cartilage contributing to enhanced neo-formation of cartilaginous tissue in vivo.

Guillermo Bauza-*Mayol*[1,2,3], *Marcos Quintela*[3], *Ava Brozovich*[1,2,4], *Michael Hopson*[2], *Shazad Shaikh*[2], *Fernando Cabrera*[1,2], *Aaron Shi*[1,2], *Federica Banche Niclot*[1,6], *Francesca Paradiso*[1,2,3], *Emma Combellak*[5], *Tom Jovic*[5], *Paul Rees*[2], *Ennio Tasciotti*[1,2,7], *Lewis W Francis*[1]*, *Patrick Mcculloch*[2], *Francesca Taraballi*[1,2]*.

Supplementary Materials

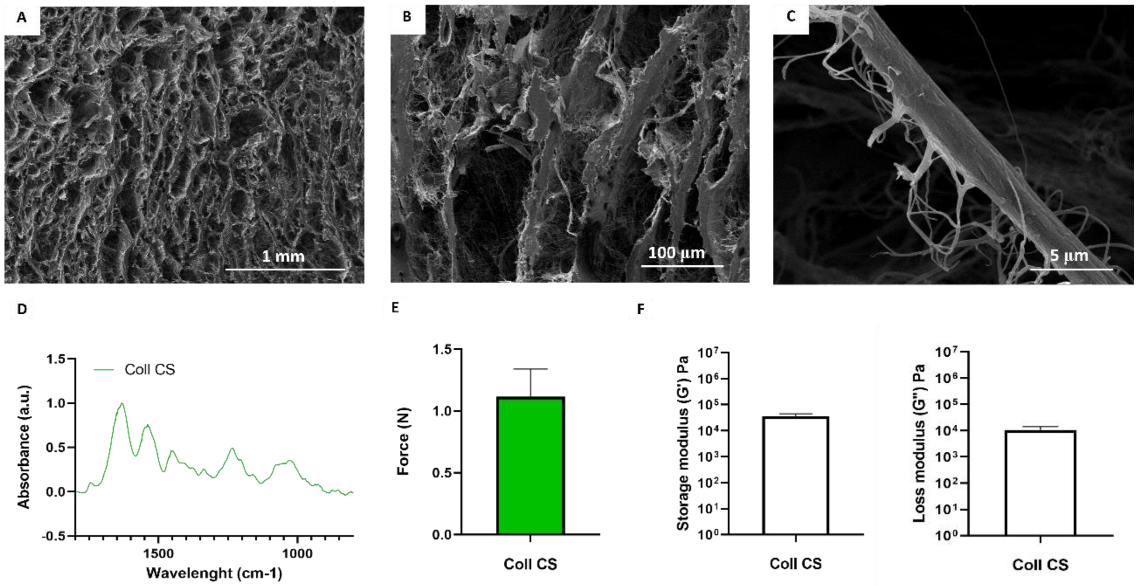

Supplementary Figure 1. (S1)

Supplementary Figure 1. Structural and chemical characterization of the CLCS scaffolds. (A-B-C) Scanning electron microscope images of CSCL at different magnification. Scale bars are included in the figure. (D) Fourier transform infrared

spectroscopy spectra of CSCL. The spectra highlighted the presence of typical collagen Amide I, Amide II, Amide III. The presence of CS inside the collagen scaffold represented mainly on the sugar peaks that arise around 1000 cm -1. (E) Compression test performed on CSCL scaffolds. Data are mean 3 + standard dev (SD). (F) Rheology analysis of storage modulus (G', Pa) and loss modulus (G", Pa) in CSCL scaffolds. Data are mean 3 + standard dev (SD).

Supplementary material S2. List of differentially expressed genes in untreated cartilage defects in comparison to healthy control at 12 weeks post-surgery (FDR < 0.05, p<0.05).

Gene name	log2FoldChange	Fold change	P adj.
FBXL13	3,9439	15,3903	0,00000
POSTN	3,7271	13,2421	0,00000
MMP13	3,6959	12,9595	0,00000
LUM	3,6801	12,8177	0,00000
PMCH	3,4694	11,0763	0,00000
COL1A2	3,3098	9,9164	0,00000
SLC9A5	3,3055	9,8869	0,00000
COL1A1	3,2740	9,6732	0,00000
OIP5	3,2618	9,5921	0,00000
CRYBG2	3,0838	8,4783	0,00000
SLC19A1	3,0742	8,4222	0,00000
CERKL	3,0548	8,3096	0,00000
METTL7B	3,0109	8,0607	0,00000
EPG5	2,8768	7,3451	0,00000
DUOXA2	2,8283	7,1024	0,00000
KCNH4	2,8283	7,1024	0,00002
KIAA0895	2,7010	6,5024	0,00080
CLEC12B	2,6223	6,1571	0,00001
METTL4	2,6168	6,1341	0,00061
B4GALNT1	2,5964	6,0480	0,00006
SLC14A1	2,5922	6,0300	0,00009
KCTD19	2,5919	6,0290	0,00204
HMGXB3	2,5787	5,9741	0,00000
MROH2B	2,5697	5,9367	0,00003
CCDC152	2,5541	5,8730	0,00000
ZSWIM1	2,5371	5,8044	0,00140
C12orf4	2,5154	5,7177	0,00563

CTSK	2,5006	5,6593	0,00002
TBATA	2,4887	5,6127	0,00863
NUDCD2	2,4327	5,3990	0,00693
MRM3	2,4244	5,3680	0,00750
SLC1A7	2,4144	5,3308	0,00928
MMP9	2,4051	5,2967	0,00004
FLAD1	2,3981	5,2711	0,00008
SLC15A3	2,3388	5,0588	0,00000
DDX39A	2,3373	5,0537	0,00000
SCRN3	2,3046	4,9402	0,00000
ZWILCH	2,2984	4,9192	0,01761
THBS2	2,2737	4,8355	0,01043
MARCKSL1	2,2707	4,8255	0,02084
SPNS1	2,2597	4,7888	0,00856
CTSA	2,2334	4,7025	0,02204
BCKDHB	2,2318	4,6971	0,00314
NCOA5	2,2107	4,6291	0,00008
APOA2	2,2092	4,6242	0,02183
MROH2A	2,2076	4,6190	0,00193
YARS	2,1964	4,5832	0,00270
LMF2	2,1932	4,5731	0,00008
PRMT7	2,1852	4,5479	0,00039
C1QL3	2,1788	4,5279	0,00000
SEMA6D	2,1588	4,4654	0,00998
DSG1	2,1491	4,4356	0,00180
ATP6V0D2	2,1475	4,4306	0,02639
IQGAP2	2,1311	4,3807	0,00340
ADCY3	2,1283	4,3721	0,00000
5S_rRNA	2,1265	4,3667	0,02247
UBE2O	2,1248	4,3615	0,00054
PPIE	2,1065	4,3065	0,00000
IBSP	2,0882	4,2522	0,00000
SPHK1	2,0708	4,2011	0,01130
PRICKLE2	2,0607	4,1720	0,04488
PROX2	2,0441	4,1243	0,00126
OLFML2B	2,0410	4,1154	0,01119
TESK1	1,9975	3,9930	0,00247
EIF2AK4	1,9914	3,9761	0,00000
MMP2	1,9896	3,9713	0,02774
TTLL5	1,9858	3,9609	0,00000
SYN1	1,9833	3,9538	0,00097
ENPP5	1,9618	3,8956	0,00647
SLC18A2	1,9317	3,8151	0,02134

CALD1	1,9269	3,8024	0,00329
ZCWPW1	1,9130	3,7658	0,00180
NPAS1	1,9129	3,7657	0,02662
TRPV2	1,9075	3,7517	0,01759
ZFHX2	1,9005	3,7335	0,03537
FRYL	1,8943	3,7174	0,00450
VMP1	1,8776	3,6747	0,02991
PTRHD1	1,8690	3,6527	0,02426
AMBRA1	1,8669	3,6476	0,00418
POLDIP2	1,8336	3,5643	0,00000
LAMTOR1	1,8205	3,5320	0,02577
MMP19	1,8187	3,5276	0,00525
BMP1	1,8022	3,4875	0,03615
TYROBP	1,7867	3,4503	0,02517
PDE6A	1,7865	3,4497	0,02042
PCYT1B	1,7786	3,4310	0,02433
APBB1IP	1,7747	3,4218	0,00856
GLI1	1,7708	3,4125	0,00170
BASP1	1,7661	3,4014	0,03835
MFSD8	1,7660	3,4010	0,02984
CLSTN1	1,7649	3,3985	0,00044
EPB41L2	1,7618	3,3912	0,03359
COPG2	1,7577	3,3815	0,04520
PITPNM3	1,7567	3,3792	0,00343
BCAS1	1,7261	3,3084	0,03023
TRPT1	1,7148	3,2825	0,00105
COMT	1,7059	3,2624	0,00040
SPP1	1,7052	3,2608	0,00153
PIGH	1,6834	3,2118	0,00003
TFRC	1,6745	3,1920	0,01811
COL4A1	1,6555	3,1504	0,01802
COL22A1	1,6521	3,1430	0,01839
SLC26A10	1,6445	3,1263	0,03436
UBA7	1,6389	3,1142	0,00033
NYAP2	1,6016	3,0349	0,01631
SKIV2L2	1,6009	3,0333	0,02240
FAM120B	1,5985	3,0284	0,00250
NEK8	1,5908	3,0121	0,00109
GFM2	1,5798	2,9893	0,00524
IL23A	1,5792	2,9881	0,02872
MTX1	1,5584	2,9453	0,00109
DKC1	1,5548	2,9380	0,00040
RNF167	1,5338	2,8955	0,00028

DNAH11	1,5233	2,8745	0,00008
PRIMPOL	1,5168	2,8615	0,00356
FAM198B	1,5140	2,8559	0,01882
SWSAP1	1,4963	2,8213	0,00133
NCAPH2	1,4903	2,8094	0,00380
TNC	1,4798	2,7891	0,03359
RXRB	1,4742	2,7782	0,01474
VPS29	1,4682	2,7668	0,00038
SMG6	1,4508	2,7336	0,01476
PAQR6	1,4252	2,6855	0,00515
TMSB10	1,4092	2,6558	0,04548
LRRC41	1,3722	2,5886	0,01303
ASTE1	1,3358	2,5242	0,00304
PKD2L2	1,3339	2,5208	0,02859
TDRD6	1,3320	2,5175	0,02563
PLTP	1,3232	2,5023	0,00049
BGLAP	1,3213	2,4988	0,00754
AUP1	1,3172	2,4918	0,04408
ELAC2	1,3134	2,4853	0,00888
PPT1	1,3026	2,4667	0,00021
DNAJC17	1,2942	2,4524	0,01802
SLC24A5	1,2916	2,4481	0,00250
AGT	1,2820	2,4318	0,03674
TMEM150B	1,2694	2,4106	0,00133
FANK1	1,2672	2,4069	0,01631
KAT5	1,2519	2,3816	0,01952
TP53TG5	1,2428	2,3666	0,02319
PPIL2	1,1995	2,2967	0,01897
SDS	1,1907	2,2827	0,01395
B4GAT1	1,1843	2,2725	0,02703
KATNA1	1,1757	2,2591	0,00983
TMEM204	1,1647	2,2418	0,01043
PPOX	1,1617	2,2372	0,00380
ERBB2	1,1525	2,2230	0,00563
FAM227B	1,1432	2,2086	0,01631
CCDC65	1,1314	2,1907	0,00452
BMP2K	1,1279	2,1854	0,04718
EDC3	1,0812	2,1158	0,00801
CAST	1,0788	2,1123	0,02839
RAD9A	1,0757	2,1078	0,01942
RHPN2	1,0679	2,0964	0,01472
SLC46A1	1,0637	2,0903	0,01631
NR1I3	1,0604	2,0855	0,04805

EMC4	1,0536	2,0757	0,01626
ETNK2	1,0420	2,0591	0,02517
HMGN5	1,0349	2,0490	0,01474
VRK2	1,0221	2,0308	0,01474
RPL10	0,9938	1,9914	0,02134
C1orf167	0,9927	1,9900	0,03750
LRRC71	0,9658	1,9531	0,01942
CRBN	0,9552	1,9388	0,04640
MYH11	0,9349	1,9118	0,02976
GPLD1	0,9155	1,8863	0,01907
ND6	0,9097	1,8787	0,03677
MAP3K15	0,9051	1,8727	0,01802
CNOT1	0,8735	1,8321	0,03296
ADAMTSL4	-0,8332	0,5613	0,04414
SPRYD3	-1,0049	0,4983	0,03813
CADM4	-1,2137	0,4311	0,01165
NCAPD2	-1,2456	0,4217	0,00833
TSC2	-1,2632	0,4166	0,01578
COPB2	-1,2644	0,4163	0,00938
BBS4	-1,3102	0,4033	0,00983
GBA3	-1,3338	0,3967	0,01180
ZNF335	-1,3345	0,3965	0,02774
DDX17	-1,3405	0,3949	0,00971
ZNF292	-1,3506	0,3921	0,01183
AP2A1	-1,3544	0,3911	0,00998
FHOD1	-1,3676	0,3875	0,03884
FAN1	-1,3819	0,3837	0,00133
TNS2	-1,3946	0,3803	0,00459
THBS1	-1,4040	0,3779	0,04690
SH2B3	-1,4164	0,3746	0,01921
SYTL4	-1,4246	0,3725	0,02227
MINK1	-1,4379	0,3691	0,01143
C19orf66	-1,4434	0,3677	0,02235
PCNX4	-1,4504	0,3659	0,02577
FANCC	-1,4508	0,3658	0,02043
GAPDH	-1,4534	0,3652	0,02595
UBP1	-1,4659	0,3620	0,00153
LRMP	-1,4670	0,3617	0,00485
UNC93A	-1,5324	0,3457	0,00239
DIXDC1	-1,5332	0,3455	0,00091
FMOD	-1,5373	0,3445	0,02145
IRF2BPL	-1,5440	0,3429	0,03396
WDR54	-1,5770	0,3352	0,00771

LRRC30	-1,6027	0,3293	0,00804
NKIRAS2	-1,6059	0,3285	0,02375
CTGF	-1,6095	0,3277	0,00193
HTRA2	-1,6128	0,3270	0,01043
MYO18A	-1,6187	0,3256	0,01911
SOD3	-1,6193	0,3255	0,02344
KPNA1	-1,6297	0,3231	0,00004
UBE2T	-1,6324	0,3226	0,02230
RBFOX3	-1,6389	0,3211	0,01739
BEST1	-1,6626	0,3159	0,00018
CFAP44	-1,6726	0,3137	0,02552
ANXA8	-1,6998	0,3078	0,00418
ADM	-1,7194	0,3037	0,03312
MINDY1	-1,7197	0,3036	0,01631
THBS4	-1,7299	0,3015	0,02828
5S_rRNA	-1,7563	0,2960	0,02926
SERPINA3	-1,7720	0,2928	0,01914
IFFO1	-1,7995	0,2873	0,00000
SETD1A	-1,8078	0,2856	0,00584
SMOC2	-1,8214	0,2829	0,00198
CLU	-1,8240	0,2824	0,00349
BOC	-1,8266	0,2819	0,01882
TMEM263	-1,8314	0,2810	0,00164
FAM151A	-1,8325	0,2808	0,00000
PITRM1	-1,8369	0,2799	0,00550
HSF4	-1,8548	0,2765	0,01631
SOX9	-1,8601	0,2755	0,03975
LMCD1	-1,8698	0,2736	0,02517
MTBP	-1,8966	0,2686	0,00265
THRA	-1,8983	0,2683	0,00009
VPS54	-1,9502	0,2588	0,00655
SHMT2	-1,9513	0,2586	0,00001
ENO1	-1,9723	0,2548	0,00349
S100P	-1,9858	0,2525	0,04443
ERRFI1	-2,0020	0,2497	0,00061
TLN1	-2,0322	0,2445	0,00021
SERPINE2	-2,0358	0,2439	0,00071
SSH3	-2,0465	0,2421	0,00049
LYPLA2	-2,0496	0,2415	0,00335
BARX1	-2,0511	0,2413	0,00000
SPATA46	-2,0699	0,2382	0,01373
EPHB6	-2,0982	0,2335	0,02809
KIAA1211	-2,1233	0,2295	0,00257

DCN	-2,1257	0,2291	0,00019
ITGBL1	-2,1304	0,2284	0,03214
GALNT2	-2,1405	0,2268	0,01527
KIAA0355	-2,1413	0,2267	0,00329
MTFR1	-2,1722	0,2219	0,01802
DOK1	-2,1953	0,2183	0,00601
LOXL3	-2,2033	0,2171	0,00004
HIF1A	-2,2181	0,2149	0,00001
PTGES	-2,2249	0,2139	0,02338
WWP2	-2,2355	0,2124	0,00020
GREM1	-2,2499	0,2102	0,01556
TNFRSF11B	-2,2621	0,2085	0,01520
CYTL1	-2,2656	0,2080	0,00003
LRRIQ4	-2,2687	0,2075	0,00007
TUB	-2,2974	0,2034	0,00295
ACSF2	-2,2989	0,2032	0,00001
TLR5	-2,3109	0,2015	0,01808
TSC22D2	-2,3148	0,2010	0,00596
GRIN2C	-2,3362	0,1980	0,01658
CSNK1D	-2,3631	0,1944	0,00000
NUP54	-2,3695	0,1935	0,01165
TAPBPL	-2,3776	0,1924	0,00000
ANKRD13D	-2,3986	0,1897	0,00448
TNIP1	-2,4295	0,1856	0,00007
PPP1R1B	-2,4378	0,1846	0,00849
DACT2	-2,4430	0,1839	0,00424
NYAP1	-2,4521	0,1827	0,00276
FZD9	-2,4854	0,1786	0,00693
TMEM184B	-2,4993	0,1769	0,00000
SEMA3D	-2,5559	0,1701	0,00016
G0S2	-2,5568	0,1700	0,00642
TMIE	-2,6091	0,1639	0,00340
MRS2	-2,6119	0,1636	0,00126
C2orf40	-2,6162	0,1631	0,00040
CILP	-2,6212	0,1625	0,00000
OGN	-2,6564	0,1586	0,00004
PPP1R3C	-2,6639	0,1578	0,00011
ANGPTL7	-2,6728	0,1568	0,00004
SCN5A	-2,6802	0,1560	0,00302
AGFG2	-2,7088	0,1530	0,00051
ALKBH5	-2,7332	0,1504	0,00008
STC2	-2,7370	0,1500	0,00000
BARHL1	-2,8065	0,1429	0,00170

AP5Z1	-3,0084	0,1243	0,00000
M1AP	-3,0442	0,1212	0,00012
IL21	-3,0510	0,1207	0,00009
SLC38A4	-3,0622	0,1197	0,00000
CA9	-3,0951	0,1170	0,00009
UVRAG	-3,1947	0,1092	0,00025
NUDT6	-3,3552	0,0977	0,00000
TPR	-4,0822	0,0590	0,00000

Supplementary S3.

Supplementary materials S3. List of differentially expressed genes in CLCS treated cartilage defects in comparison to healthy control at 12 weeks post-surgery (FDR < 0.05, p<0.05).

Gene name	log2FoldChange	Fold change	P adj.
FBXL13	3,71763839	13,1559031	6,657E-12
POSTN	3,68950101	12,901805	1,0249E-09
COL1A1	3,35020397	10,1979267	5,8645E-13
METTL7B	3,23173701	9,39398316	6,0956E-10
LUM	3,17504091	9,03197137	3,2669E-05
COL1A2	3,14219043	8,82863519	2,1079E-14
SLC9A5	2,99592314	7,97742498	9,9272E-07
C12orf4	2,94299687	7,69007075	0,00074886
OIP5	2,9413608	7,68135488	4,0901E-07
PMCH	2,79898582	6,95951044	4,2188E-08
MROH2B	2,77409249	6,84045593	6,2026E-06
SLC19A1	2,70623794	6,52617622	3,0376E-07
KIAA0895	2,69813627	6,48963018	0,00098725
MMP13	2,67270138	6,37621986	4,6021E-06
CRYBG2	2,64556602	6,25741168	0,000176
METTL4	2,60086109	6,06648603	0,00085757
MMP9	2,53227013	5,78481224	1,7107E-05
B4GALNT1	2,49359121	5,63178091	0,000176
KCTD19	2,39644021	5,26502438	0,0068016
HMGXB3	2,36978663	5,16864685	5,4459E-11
DDX39A	2,34565421	5,08290831	1,6995E-09
COL22A1	2,32256297	5,00220079	0,00020459
PRMT7	2,31120765	4,96298347	0,000176
DUOXA2	2,2858242	4,87642613	0,00074886
IBSP	2,23681137	4,71354129	7,7707E-07
SLC15A3	2,22220045	4,66604573	2,9008E-08

SCRN3	2,20345096	4,60579742	3,0376E-07
FLAD1	2,19828563	4,58933662	0,00054538
COPG2	2,18784031	4,55622916	0,00777267
TESK1	2,17297718	4,50953032	0,00082532
SPNS1	2,13696873	4,39836927	0,01904741
LMF2	2,13350955	4,38783583	0,000176
MARCKSL1	2,12517754	4,36256776	0,04515582
CLEC12B	2,0719087	4,20442554	0,00184947
EPB41L2	2,06624634	4,18795616	0,00998347
KCNH4	2,0536641	4,15159037	0,00766742
CALD1	2,0428629	4,12062422	0,00184947
BMP1	2,0270577	4,07572778	0,01719344
OLFML2B	2,02334459	4,06525144	0,01564072
ADCY3	1,99233879	3,97881491	3,6501E-06
COL4A1	1,97861832	3,94115454	0,00325045
PPIE	1,97421442	3,92914231	4,0775E-05
TRPV2	1,94621663	3,85362618	0,01859391
COMT	1,9441778	3,84818405	3,8781E-05
DSG1	1,93421946	3,82171304	0,00888739
CERKL	1,88243771	3,68697519	3,7194E-06
EIF2AK4	1,86619699	3,64570288	3,5338E-08
SLC14A1	1,86493676	3,64251967	0,01749095
MROH2A	1,85711046	3,6228133	0,01859391
SLC26A10	1,84232287	3,5858692	0,01695623
POLDIP2	1,82967178	3,55456194	4,0124E-06
PAQR6	1,81619641	3,52151545	0,00014786
EPG5	1,81252412	3,51256306	0,0087413
NCOA5	1,8039086	3,49164916	0,00384528
TTLL5	1,79229414	3,46365236	7,5979E-07
CCDC152	1,78675152	3,45037106	0,00467881
FRYL	1,78414625	3,44414588	0,01193758
HIPK1	1,77108027	3,41309429	0,01326829
CTSK	1,73577008	3,33057225	0,01193758
UBA7	1,73400423	3,32649814	0,00014968
SWSAP1	1,73185845	3,32155419	0,00013631
PROX2	1,73121412	3,32007105	0,01330935
COL4A2	1,7168852	3,28725916	0,01695623
BGLAP	1,71575797	3,28469171	0,000176
UBE2O	1,70543994	3,26128368	0,01385779
ELAC2	1,69246598	3,23208687	0,00027961
RXRB	1,64731716	3,13250576	0,00567472
NCAPH2	1,61859716	3,07076298	0,00157272
SKIV2L2	1,61771218	3,06887989	0,02710255

CLSTN1	1,60519126	3,0423608	0,00258447
PITPNM3	1,58151572	2,99284118	0,01481621
GLI1	1,56570097	2,96021297	0,01033237
SPP1	1,56325628	2,95520105	0,00601209
TNC	1,53125026	2,89036214	0,0334512
AMBRA1	1,53054053	2,88894058	0,04029148
MMP19	1,52206897	2,87202632	0,03910048
FAM120B	1,50887586	2,84588203	0,00675819
SDS	1,49783649	2,8241887	0,00090292
C1QL3	1,4831982	2,795678	0,00563989
MTX1	1,47820692	2,78602251	0,00307768
TMEM150B	1,4779178	2,78546425	0,00012416
NEK8	1,46170048	2,7543282	0,00473281
RNF167	1,43721201	2,70797048	0,00104498
DNAJC17	1,42422915	2,68371069	0,00876417
R3HCC1L	1,41767012	2,67153722	0,03149713
FAM198B	1,40486329	2,64792689	0,04503547
PLTP	1,39912857	2,63742225	0,000213
ERBB2	1,39227663	2,62492579	0,00044742
KAT5	1,38656384	2,61455214	0,00914732
DNAH11	1,36994746	2,58461153	0,00074886
RHPN2	1,33389021	2,52081495	0,0011064
PIGH	1,32818547	2,51086676	0,00317824
CCDC65	1,31803106	2,49325607	0,00061679
ARHGAP9	1,30279702	2,46706721	0,0192575
PPIL2	1,25577125	2,38794771	0,0157695
MYH11	1,25479072	2,3863253	0,00150373
BMP6	1,23841463	2,35939118	0,01482926
CYP8B1	1,23665908	2,35652188	0,00858012
SLC46A1	1,23398339	2,35215542	0,00429472
ETNK2	1,2331426	2,35078501	0,0061521
RAD9A	1,22840915	2,34308477	0,00651257
PMM1	1,22123966	2,33146967	0,01985879
PRIMPOL	1,20876011	2,31138905	0,04484638
DUOX1	1,20657517	2,30789113	0,01911171
CRBN	1,19002389	2,28156521	0,00818319
PPOX	1,17223087	2,25359906	0,004524
B4GAT1	1,14621887	2,21333046	0,04544305
DKC1	1,12089435	2,17481751	0,02970741
PPT1	1,10114337	2,1452464	0,00393972
SIMC1	1,08763626	2,12525546	0,04097304
WDR74	1,08091009	2,11537009	0,01911171
KATNA1	1,06817352	2,09677713	0,03000373

APBB1	1,06481161	2,0918967	0,03789458
STAB1	1,05419566	2,07656014	0,03000373
LRRC71	1,0329255	2,04616927	0,01330935
DDB2	0,98100239	1,97383637	0,03789458
RPL10	0,96751532	1,95546988	0,03375358
UBQLN4	0,95608368	1,94003634	0,04503547
DAGLB	0,95190411	1,93442408	0,01803709
FLOT2	0,95146382	1,93383381	0,01828116
HMGN5	0,94854105	1,92992	0,03789458
EDC3	0,93728071	1,91491547	0,03744885
SLC35A2	0,89344835	1,85761091	0,02841494
NCAPD3	0,88648159	1,84866215	0,03018535
MAP3K15	0,82671307	1,77363982	0,04635911
ADAMTSL4	-0,9112643	0,53171892	0,02732118
FAN1	-1,0825398	0,47219681	0,0264153
KPNA1	-1,0998885	0,46655256	0,01768864
IFFO1	-1,1389595	0,45408695	0,00666224
BARX1	-1,1813661	0,44093377	0,02127105
UBP1	-1,2408548	0,42312187	0,0140257
UNC93A	-1,2916514	0,4084832	0,01923772
LRMP	-1,3182591	0,40101856	0,01911171
BBS4	-1,358883	0,38988404	0,00878241
LOXL3	-1,3719505	0,38636854	0,03555601
SMOC2	-1,3814866	0,38382308	0,04170148
LRRC30	-1,3980988	0,37942883	0,03555601
SYTL4	-1,4071557	0,37705432	0,03319776
COPB2	-1,4118819	0,37582114	0,00365663
CYTL1	-1,4153329	0,37492321	0,02649462
ZNF335	-1,4346256	0,36994287	0,02100628
TPM2	-1,4455559	0,36715067	0,03312616
DIXDC1	-1,4938342	0,35506763	0,00172058
ACSF2	-1,5045896	0,35243043	0,0121643
MYO18A	-1,5059013	0,35211014	0,04504732
FAM151A	-1,5322437	0,34573926	7,8318E-06
IRF2BPL	-1,565067	0,33796202	0,04272283
MINDY1	-1,5854526	0,33322011	0,04173792
TNIP1	-1,5885421	0,33250728	0,02669956
LYPLA2	-1,6178919	0,32581121	0,04179753
PLCG1	-1,6725034	0,31370852	0,01356355
DCN	-1,6935505	0,30916512	0,00682929
PRRC2C	-1,7039398	0,30694673	0,04982189
TMEM263	-1,7065015	0,30640219	0,00542139
ERRFI1	-1,7144022	0,30472881	0,00666224

ARL9	-1,7755347	0,29208603	0,039964
VPS54	-1,7968665	0,287799	0,01911171
PPP1R3C	-1,8697603	0,27361889	0,01631995
SPATA46	-1,9014601	0,26767234	0,03575558
CSNK1D	-1,9177233	0,26467185	2,3769E-05
HIF1A	-1,9485906	0,2590692	0,00014786
AGFG2	-1,94976	0,25885928	0,02933096
SERPINE2	-1,9602945	0,256976	0,00165101
TAPBPL	-1,9646164	0,25620731	4,4987E-05
STC2	-1,9724727	0,25481591	0,00071655
SETD1A	-1,9741641	0,25451735	0,00311801
TUB	-1,9833478	0,25290233	0,01803709
AP5Z1	-1,9998839	0,25002012	0,00157272
BEST1	-2,0145767	0,24748677	4,6021E-06
SEMA3D	-2,0316938	0,24456778	0,00589727
PITRM1	-2,0732846	0,23761789	0,00176066
C2orf40	-2,0815614	0,23625857	0,0106284
PDPN	-2,1050865	0,2324373	0,02254649
DACT2	-2,1080429	0,23196147	0,02353721
TLN1	-2,1352976	0,2276205	0,00014786
LYRM4	-2,138665	0,22708983	0,03558817
5S_rRNA	-2,181666	0,22042106	0,00563989
MRS2	-2,1919166	0,21886048	0,01267037
LRRIQ4	-2,2177492	0,21497649	0,00014968
NUP54	-2,2239972	0,21404749	0,02638132
SLC38A4	-2,2267801	0,213635	3,672E-05
CILP	-2,2431828	0,21121983	2,5405E-07
NYAP1	-2,2790175	0,20603803	0,0087256
FBXO25	-2,3353836	0,19814334	0,00752038
OGN	-2,3552359	0,19543545	0,00052158
TMEM184B	-2,3705822	0,19336758	1,8867E-09
CLU	-2,3906648	0,19069451	5,3124E-05
TPR	-2,4785889	0,17941981	3,2726E-09
IL21	-2,5365555	0,17235374	0,0024698
ANGPTL7	-2,5528756	0,17041502	0,00013817
PPP1R1B	-2,6603418	0,1581821	0,00442027
CA9	-2,8893159	0,13496751	0,00046116
M1AP	-2,932684	0,1309707	0,00035059
NUDT6	-3,0260442	0,12276369	9,1814E-15
TMIE	-3,1602916	0,11185552	0,00027961
SCN5A	-3,2686145	0,10376454	0,00017656
UVRAG	-3,3265145	0,0996826	0,00015823

Supplementary S4.

Supplementary material S4. Inflammatory pathway comparison. Diagram displaying relevant inflammatory molecular axes differentially regulated in CLCS treated cartilage compared to untreated repaired cartilage. Significant upregulated (red) or downregulated (green) RNA expression is color-coded derived from the DEseq2 across the RNA-seq data from CLCS treated and untreated cartilage compared to healthy control samples. Cut off for significance is set at *p*>0.05, log2 FC (-1,1) and FDR <0.25.

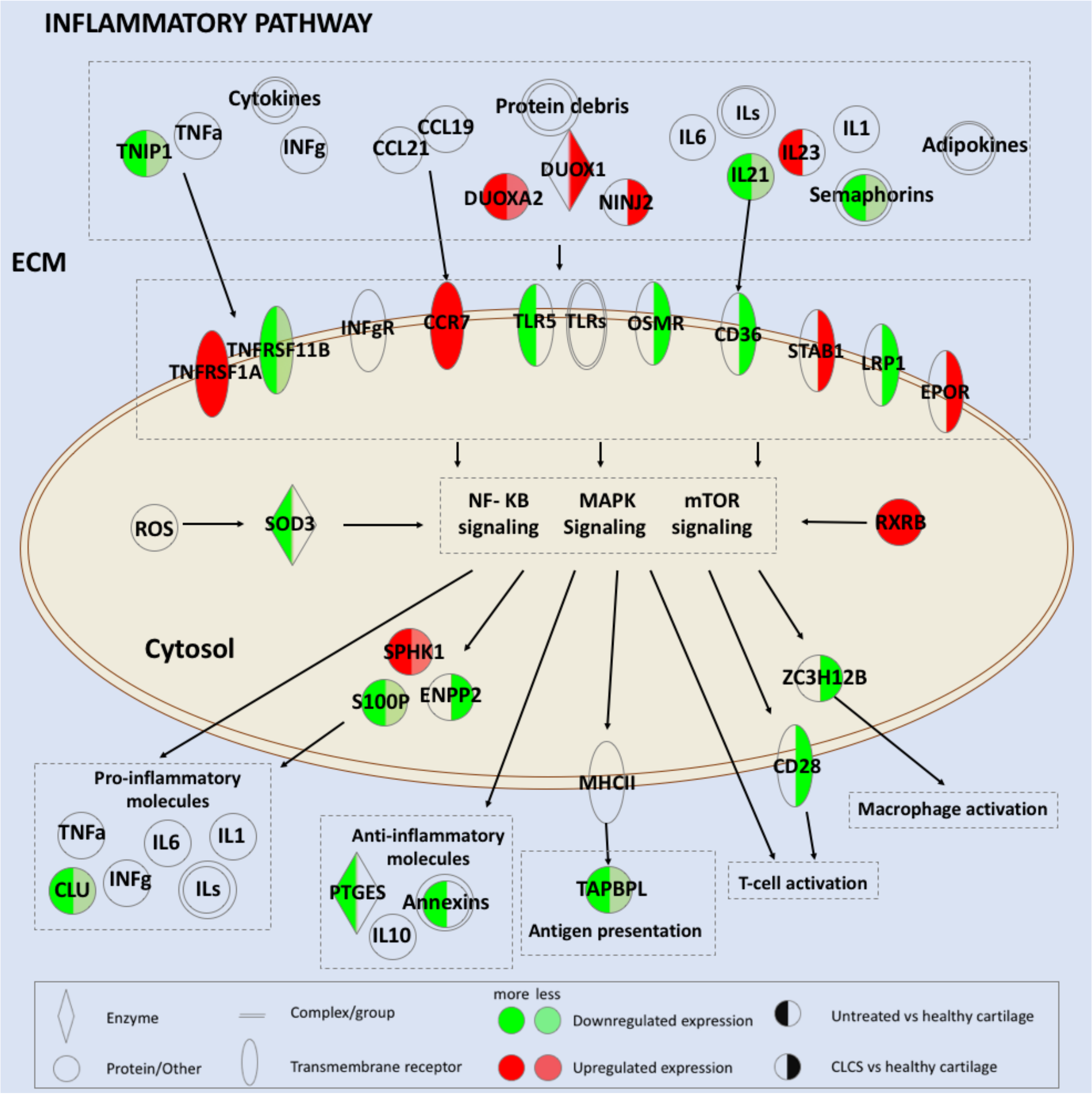

Supplementary S5.

Supplementary material S5. ECM-Cell communication pathway comparison. Diagram displaying the cellular location of relevant molecular axes involved in ECM cell interaction, focal adhesions and cell-cell interactions. Significant upregulated (red) or downregulated (green) RNA expression is color-coded derived from the DEseq2 across the RNA-seq data from CLCS treated and untreated cartilage compared to healthy control samples. Cut off for significance is set at *p*>0.05, log2 FC (-1,1) and FDR <0.25.

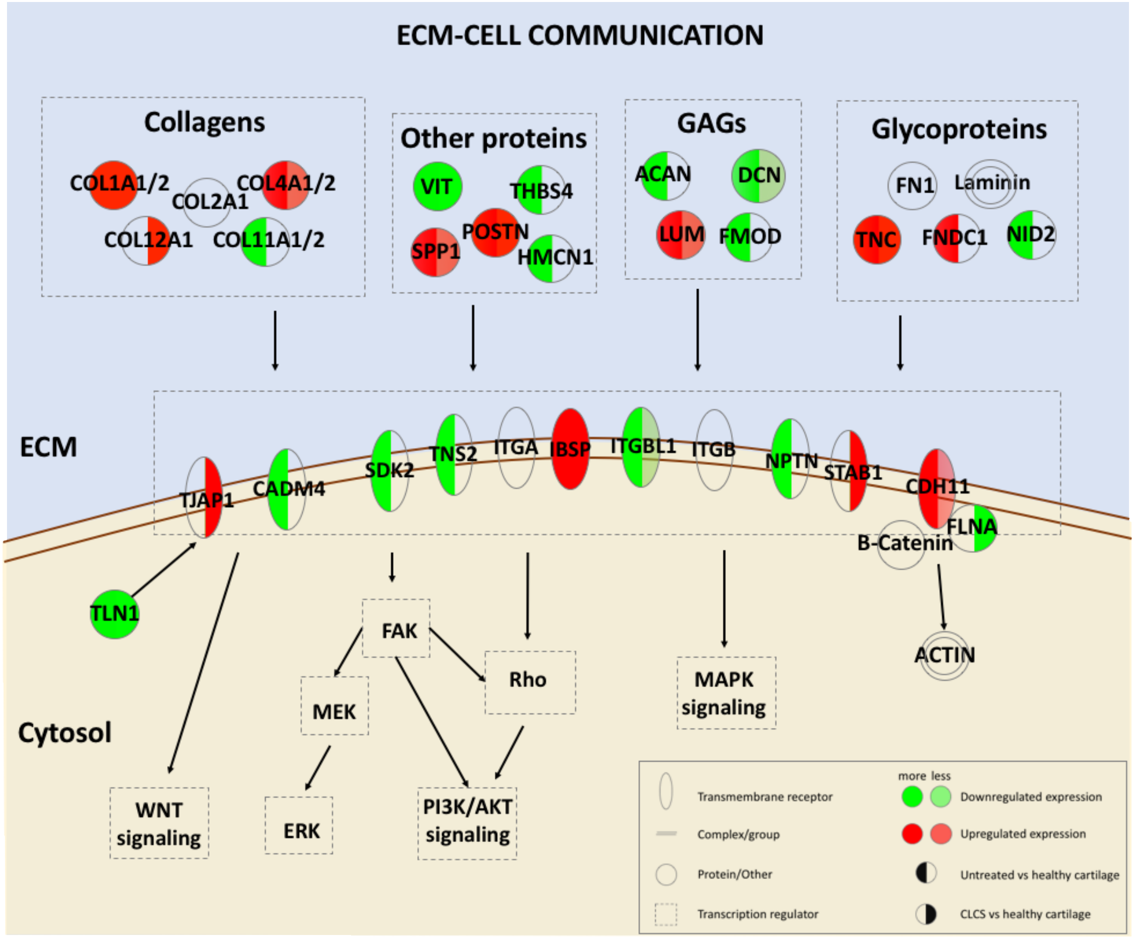

Supplementary S6.

Supplementary material S6. Cartilage homeostasis pathway comparison. Diagram displaying relevant molecular axes involved in cartilage homeostasis leading to healthy and hypertrophic cartilage ECM production. Significant upregulated (red) or downregulated (green) RNA expression is color-coded derived from the DEseq2 across

the RNA-seq data from CLCS treated and untreated cartilage compared to healthy control samples. Cut off for significance is set at *p*>0.05, log2 FC (-1,1) and FDR <0.25.

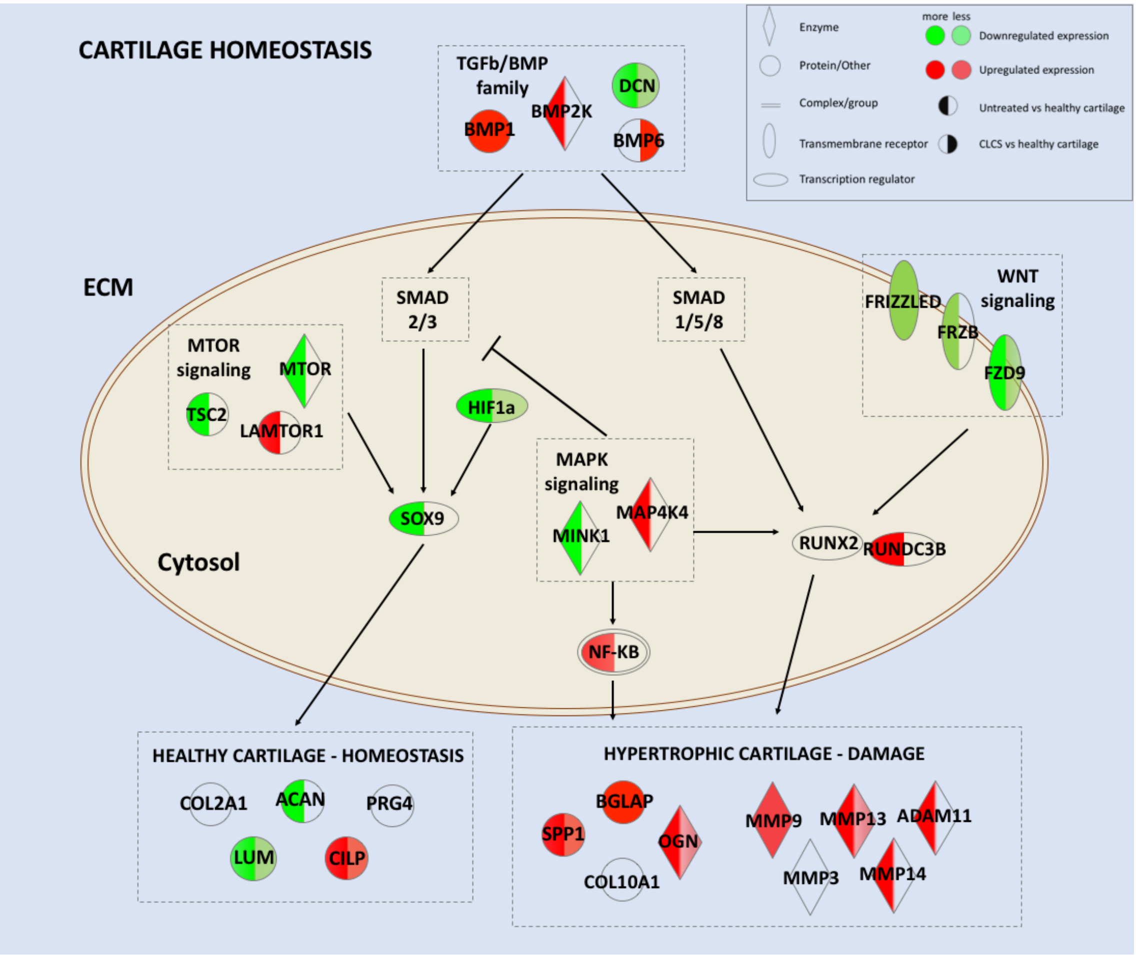

Supplementary S7.

Supplementary material S7. Articular cartilage gross anatomy regeneration. Rabbit femoral heads macroscopical images at 1-, 6- and 12-weeks post-treatment (CLCS) compared to untreated (control).

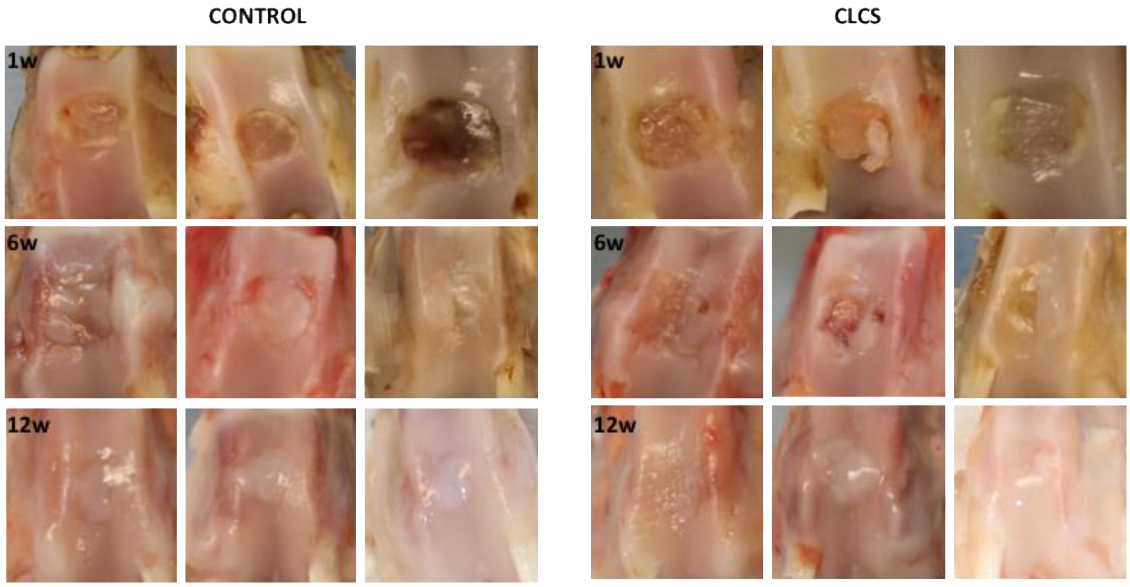

Supplementary S8.

Supplementary material S8. Articular cartilage tissue regeneration. Hematoxylin and eosin staining of rabbit *ex vivo* slides at 1-, 6- and 12-weeks post-treatment (CLCS) compared to untreated (control).

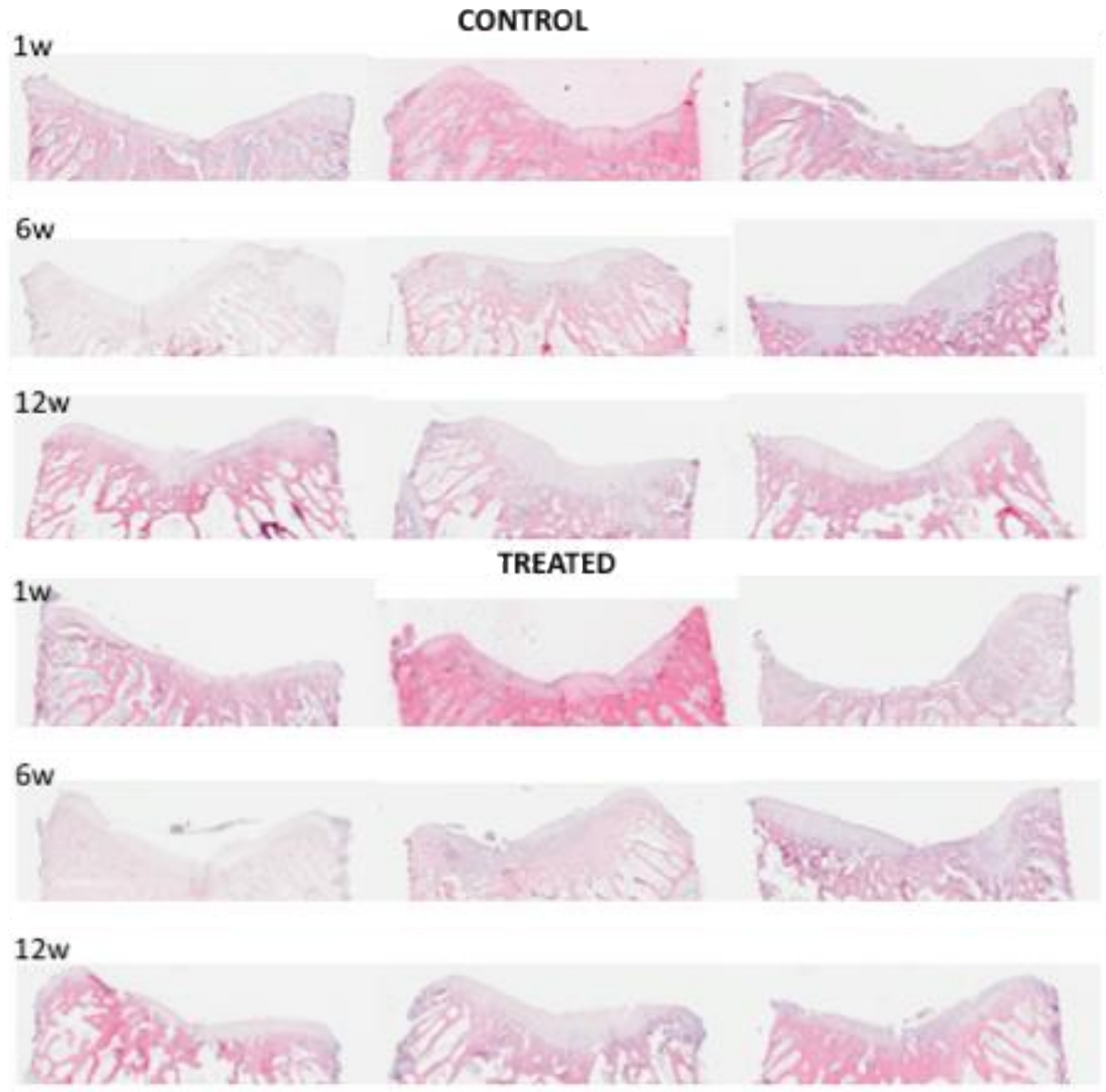
CONTROL
1w
6w
12w
TREATED
1w
6w
12w
